# Genetic Diagnosis of Adult Hemodialysis Patients With Unknown Etiology

**DOI:** 10.1016/j.ekir.2024.01.027

**Published:** 2024-02-14

**Authors:** Takuya Fujimaru, Takayasu Mori, Motoko Chiga, Shintaro Mandai, Hiroaki Kikuchi, Fumiaki Ando, Yutaro Mori, Koichiro Susa, Yuta Nakano, Takao Shoji, Yuichiro Fukudome, Naoto Inaba, Kenichiro Kitamura, Taichi Nakanishi, Keiko Uchida, Toshihiro Kimura, Teiichi Tamura, Kiyoshi Ozawa, Shinichi Uchida, Eisei Sohara

**Affiliations:** 1 Department of Nephrology, Graduate School of Medical and Dental Sciences, Tokyo Medical and Dental University, Tokyo, Japan; 2 Clinical Laboratory, Tokyo Medical and Dental University (TMDU) Hospital, Tokyo Japan; 3 Miura Seaside Clinic, Miura, Japan; 4 Zushi Sakurayama Clinic, Zushi, Japan; 5 Kitakurihama Takuchi Clinic, Yokosuka, Japan; 6 Yokosuka Clinic, Yokosuka, Japan

**Keywords:** chronic kidney disease, genetic analysis, hemodialysis, inherited kidney disease

## Abstract

**Introduction:**

Kidney disease of unknown etiology accounts for 1 in 10 adult end-stage renal disease (ESRD) cases worldwide. The aim of this study is to clarify the genetic background of patients with chronic kidney disease (CKD) of unknown etiology who initiated renal replacement therapy (RRT) in adulthood.

**Methods:**

This is a multicenter cross-sectional cohort study. Of the 1164 patients who attended 4 dialysis clinics in Japan, we first selected patients who started RRT between the ages of 20 and 49 years. After excluding patients with apparent causes of CKD (e.g., diabetic nephropathy, polycystic kidney disease (PKD) with family history, patients who underwent renal biopsy), 90 patients with CKD of unknown cause were included. The 298 genes associated with CKD were analyzed using capture-based targeted next-generation sequencing.

**Results:**

Of the 90 patients, 10 (11.1%) had pathogenic variants in CKD-causing genes and 17 (18.9%) had variant of unknown significance (VUS). Three patients had *PKD1* pathogenic variants, and 1 patient had *PKD1* and *COL4A4* pathogenic variants. In addition, 2 patients were diagnosed with atypical hemolytic uremic syndrome (aHUS) due to *C3* or *CFHR5*. One patient each was diagnosed with Alport syndrome due to *COL4A4* and *COL4A3* variants*,* nephronophthisis due to *NPHP1* variants, Fabry disease due to *GLA* variants, and autosomal-dominant tubulointerstitial kidney disease due to *UMOD* variants. Genetic diagnoses were not concordant with clinical diagnoses, except for patients with *PKD1* variant.

**Conclusion:**

This largest study on genetic analysis in hemodialysis-dependent adults revealed the presence of undiagnosed inherited kidney diseases.

## Graphical abstract

**Figure:**
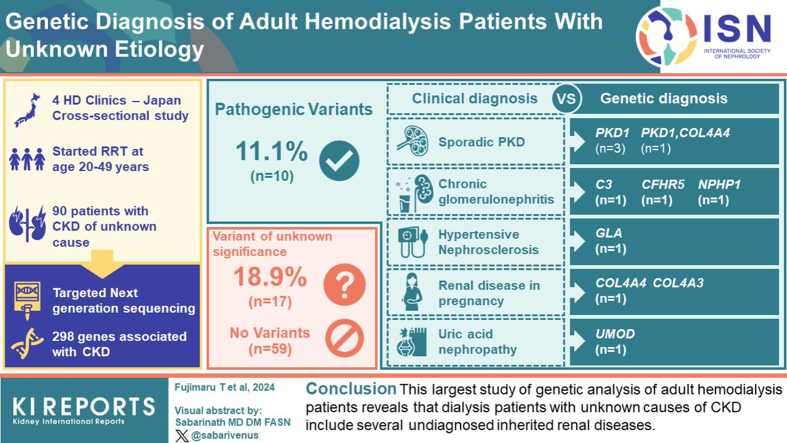

CKD affects >10% of the global population; this corresponds to >800 million individuals.^1^ In 2017, an estimated 3.9 million persons with kidney failure worldwide were treated with RRT.^2^ Hemodialysis is the most common form of RRT worldwide, accounting for approximately 69% of all cases of RRT and 89% of all dialysis modalities.^3^ Furthermore, CKD is the leading cause of mortality worldwide.^4^ CKD in adults is most commonly caused by diseases that are noninherited, such as diabetes, hypertension, and chronic glomerulonephritis (CGN).^5^ However, advancements in genetic analysis techniques have revealed that approximately 10% to 15% of adults with CKD have inherited kidney disease.^6^

Even in adults with ESRD due to hereditary kidney disease, the clinical diagnosis is inaccurate. In 2018, a Dutch study on 5 international cohorts reported that 26 of 5606 patients (0.5%) with adult-onset ESRD showed homozygous *NPHP1* deletions and were genetically diagnosed with nephronophthisis; however, only 3 (12%) were correctly diagnosed as having nephronophthisis, and the rest were misdiagnosed as having other kidney diseases or were diagnosed as having CKD with an unknown etiology.^7^ Another study on the genetic analysis of patients with CKD reported that >17% of patients with CKD with an unknown etiology had inherited kidney disease.^8^ Furthermore, kidney disease of unknown etiology accounts for 1 in 10 adult ESRD cases worldwide.^9^ These findings indicate that monogenic diseases account for a significant proportion of adult cases of CKD. A substantial number of these patients have received a nonspecific or incorrect diagnosis or a diagnosis of CKD of unknown etiology, which precludes the correct treatment, follow-up, and genetic counselling.^10^

Recently, the treatments for hereditary kidney disease have evolved. The progression of several inherited kidney diseases, such as Fabry disease and aHUS, to CKD may be prevented.^11^^,^^12^ Furthermore, previous studies have demonstrated that the inhibition of the renin–angiotensin system reduces proteinuria and decreases the rate of glomerulosclerosis and disease progression in patients with Alport syndrome.^13^^,^^14^ Moreover, a cohort study of nephronophthisis suggested that the use of angiotensin-converting enzyme inhibitors was an independent risk factor associated with early-onset ESRD in patients with pathological variants of *NPHP1*.^15^ Therefore, accurately diagnosing inherited kidney diseases may delay CKD progression and avoid RRT. Furthermore, in inherited kidney diseases with extrarenal complications, such as Fabry disease, autosomal dominant polycystic kidney disease (ADPKD), and nephronophthisis, an accurate genetic diagnosis is important for the early evaluation and prevention of complications.

A significant proportion of patients with CKD but without a specific diagnosis who require RRT may be suffering from undiagnosed hereditary kidney disease,^10^ although the exact proportion is unknown. Furthermore, by understanding the genetic causes of ESRD, we can identify genetic diseases that should be the focus of attention. To elucidate the current state of hereditary kidney disease in adults with ESRD, a large cohort study of patients on maintenance hemodialysis with unknown or undiagnosed causes is needed. Several studies have conducted genetic analyses only on patients who have undergone kidney transplantation or on patients awaiting kidney transplantation.^16^^,^^17^ In addition, a previous study that conducted genetic analysis on patients on hemodialysis lacked clinical information such as genetic diagnosis breakdown and family history.^8^ Therefore, it is necessary to accurately determine the proportion of adults with inherited kidney disease among those on maintenance hemodialysis. In our study, a comprehensive genetic analysis of 298 genes associated with CKD was conducted in patients who initiated RRT in adulthood. This study aimed to determine the proportion of patients with CKD with latent inherited kidney disease and assist in selecting those who may benefit from genetic analysis.

## Methods

### Patients

This was a multicenter cross-sectional cohort study. We investigated the data of outpatients on hemodialysis who were attending 4 dialysis clinics in Kanagawa Prefecture, Japan, in November 2019. We used the following procedure to select adult patients without an apparent cause of CKD. First, patients who started RRT between the ages of 20 and 49 years were selected, because patients who started RRT at age 50 or older have a lower incidence of inherited kidney disease.^7^^,^^17^ Second, patients with CKD with an apparent cause were defined as follows: patients with diabetic nephropathy who exhibited a typical clinical course, such as patients exhibiting a long interval from the diagnosis of diabetes to the start of dialysis, and patients with proteinuria or retinopathy, glomerular disease confirmed by renal biopsy, PKD with a positive family history, chronic pyelonephritis, renal or urinary tract tumor, and amyloid nephropathy. We then excluded patients with CKD with these apparent causes. Ultimately, the patients remaining after these selection steps were defined as having CKD of unknown etiology.

Genetic analysis was performed on all patients included in the study who provided consent. The clinical data of the patients were collected from their medical records. We extracted information on biological sex, age at initiation of RRT, age and duration of RRT at genetic analysis, and clinical diagnosis. According to the research protocol, the results of the genetic analysis were not to be communicated to the research participants, in principle. This study was approved by the research ethics committee of Tokyo Medical and Dental University.

### Genetic Analysis

Comprehensive genetic testing was performed using capture-based targeted next-generation sequencing. Overall, 298 genes related to CKD, including PKD, nephronophthisis-related ciliopathies, autosomal dominant tubulointerstitial kidney disease (ADTKD), focal segmental glomerulosclerosis, Alport syndrome, and aHUS were analyzed (Supplementary Table S1). The detailed methods are described in the Supplementary Methods and in previous reports.^18^, ^19^, ^20^ After filtering variants, all included variants were evaluated by the American College of Medical Genetics and Genomics/Association for Molecular Pathology guideline,^21^ and we defined “pathogenic” or “likely pathogenic” variants as pathogenic variants. In addition, we extracted variants with allele frequencies of <0.001 or null, a Combined Annotation-Dependent Depletion score^22^ of >20 or null, and those classified as VUS by the American College of Medical Genetics and Genomics/Association for Molecular Pathology guidelines.^21^ Among the extracted variants, a heterozygous variant in a disease with an autosomal dominant inheritance form was designated as a VUS. Moreover, we extracted heterozygous truncation variants in the inherited form of autosomal recessive (AR) disease.

To detect large genomic rearrangements, including gross deletions or duplications, copy number variation analysis was conducted using Copy Number Analysis for Targeted Resequencing (http://contracnv. sourceforge.net/).^23^ In addition, to reveal entire homozygous deletions of *NPHP1*, we performed polymerase chain reaction for exons 1, 10, and 20 of *NPHP1*.^20^ The primer sequences are shown in Supplementary Table S2.

### Statistical Analysis

The Mann–Whitney U-test was used to compare the median of continuous variables, and Fisher exact test was used to compare the percentage of categorical variables. Significance was defined as a *P*-value <0.05. All analyses were conducted using RStudio version 4.2.0. (RStudio Team [2020]; RStudio: Integrated Development for R. RStudio, PBC, Boston, MA URL http://www.rstudio.com/).

## Results

### Eligible Patients

**Figure 1:**
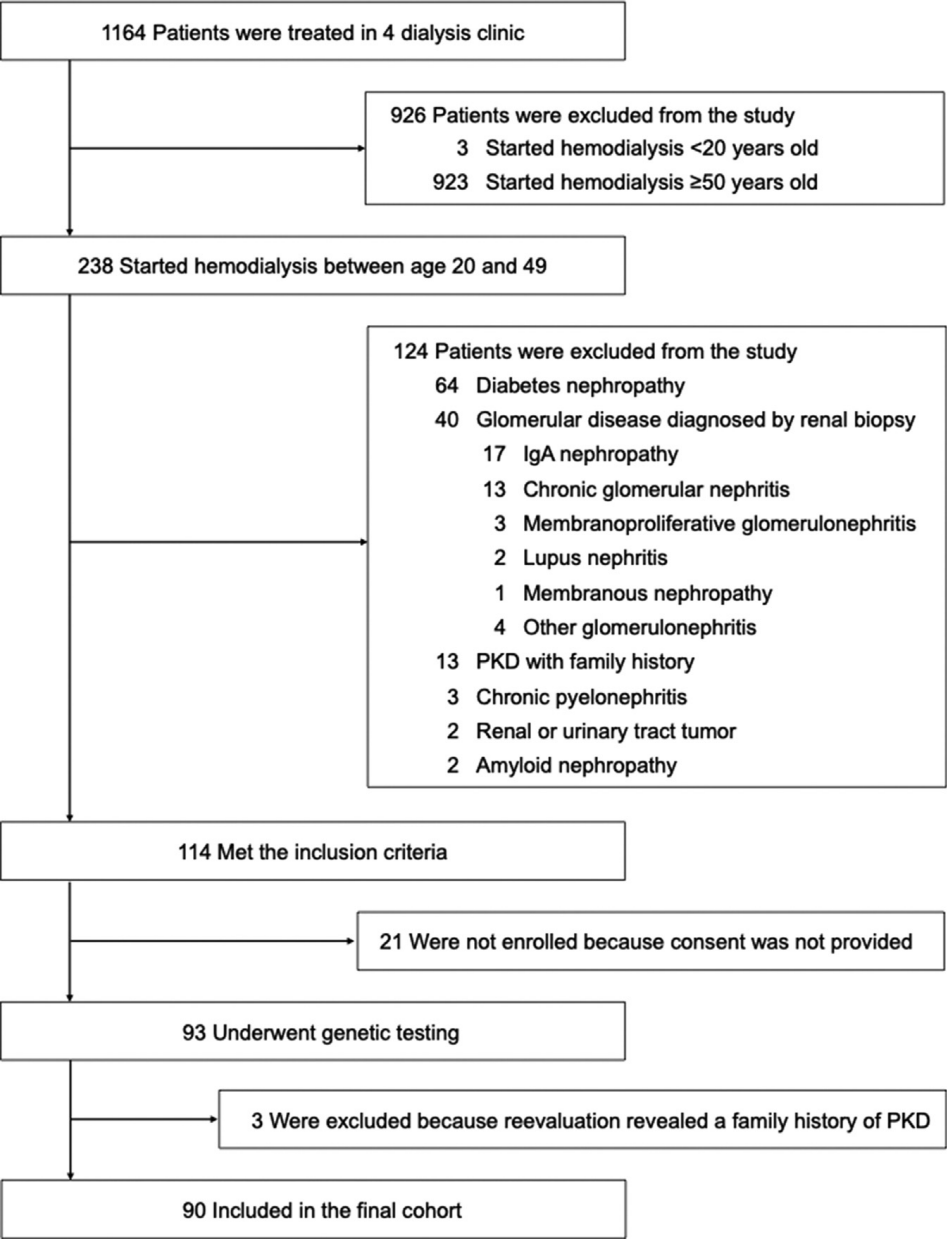
Flow chart of patient selection. Of the 1164 patients treated in 4 dialysis clinics, 283 started hemodialysis between the ages of 20 and 49 years. Of the 283 patients, 114 patients met the inclusion criteria, and 93 underwent genetic analysis. After genetic analysis and reevaluation of the medical history, 3 patients with a genetic diagnosis of autosomal dominant polycystic kidney were found to have a family history of polycystic kidney disease and were excluded from the final cohort. PKD, polycystic kidney disease.

As of November 2019, 1164 patients were attending 4 dialysis clinics in Kanagawa Prefecture, Japan. Of these, 926 patients were excluded because of the following reasons: 3 initiated dialysis at age before 20 years, and 923 initiated dialysis at age 50 years or older. Of the 238 individuals who initiated dialysis between the ages of 20 and 49 years, we excluded 124 patients with a definitive cause of CKD as follows: 64 had a clinical course typical of diabetic nephropathy, including a long period from diagnosis of diabetes to initiation of dialysis or concomitant with proteinuria and/or retinopathy; 40 had glomerular disease confirmed by renal biopsy; 13 had PKD with a positive family history; and 7 had other definitive causes of CKD. Overall, 114 patients were included in this study, and genetic analysis was conducted on 93 patients who provided consent. After genetic analysis, the medical and family histories of the patients with pathogenic variants were reevaluated, and it was found that 1 of the parents of 3 of the patients with pathologic variants in *PKD1* had PKD. Therefore, these 3 patients were excluded, bringing the final cohort to 90 patients (Figure 1). In Table 1, we list the clinical characteristics of the eligible patients. Among these patients, 51 patients (56.7%) were men, and the median (interquartile range) age at the start of dialysis was 41.0 (35.3–46.0) years. Regarding the clinical diagnosis, 46 patients (51.1%) had CGN unconfirmed by renal biopsy, 20 (22.2%) had hypertensive nephrosclerosis, 10 (11.1%) had renal disease in pregnancy, 7 (7.8%) had PKD with a negative family history, 1 (1.1%) had unilateral renal agenesis, 1 (1.1%) had renal dysplasia, 1 (1.1%) had uric acid nephropathy, and 4 (4.4%) had an unknown cause. Two of the patients with CGN had nephrotic syndrome. RRT was initiated in 4 out of the 90 patients (4.4%) aged 20 to 24 years and in 2 (2.2%) of the patients aged 25 to 29 years.

**Table 1:** Clinical characteristics of eligible patients

Clinical characteristics	All patients *N* = 90	Patients with pathogenic variant *n* = 10	Patients without pathogenic variant *n* = 80	*P*-value
Male	51 (56.7)	4 (40.0)	47 (58.8)	0.32
RRT initiation age	41.0 (35.3–46.0)	37.0 (32.0–43.0)	41.0 (36.0–46.0)	0.14
Age at genetic analysis	63.0 (51.4–69.6)	57.6 (47.2–67.1)	63.0 (52.2–69.7)	0.30
RRT duration, yrs	20.5 (10.2–29.6)	18.8 (12.0–27.8)	20.8 (10.0–29.8)	0.87
Clinical diagnosis				0.007
CGN without renal biopsy	46 (51.1)	3 (30.0)	43 (53.8)	
HNS	20 (22.2)	1 (10.0)	19 (23.8)	
Renal disease in pregnancy	10 (11.1)	1 (10.0)	9 (11.2)	
PKD without family history	7 (7.8)	4 (40.0)	3 (3.8)	
Unilateral renal agenesis	1 (1.1)	0 (0.0)	1 (1.2)	
Renal dysplasia	1 (1.1)	0 (0.0)	1 (1.2)	
Uric acid nephropathy	1 (1.1)	1 (10.0)	0 (0.0)	
Unknown	4 (4.4)	0 (0.0)	4 (5.0)	

CGN, chronic glomerulonephritis; HNS, hypertensive nephrosclerosis; PKD, polycystic kidney disease; RRT, renal replacement therapy.

Values are median (interquartile range) or number (%).

### Genetic Diagnosis

**Figure 2:**
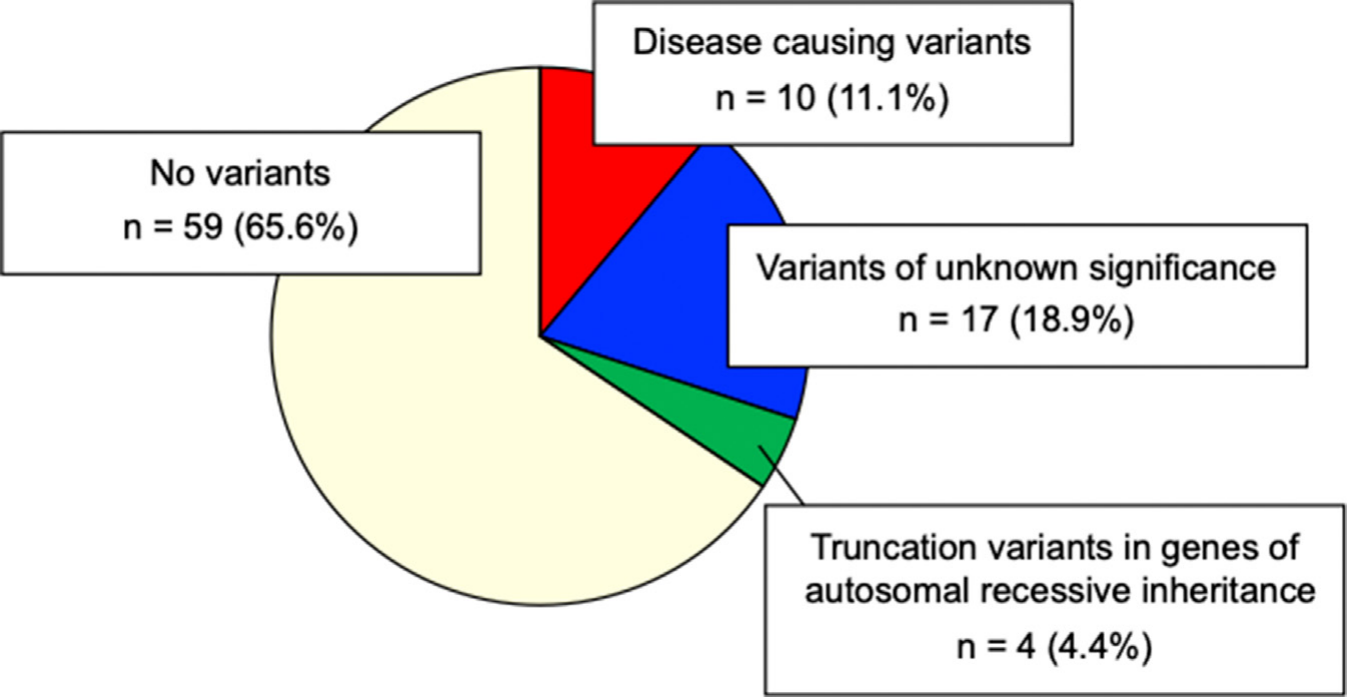
Variants in patients who started hemodialysis between the ages of 20 and 49 years. Of the 90 patients, 10 (11.1%) had disease-causing variants and 17 (18.9%) had variants of unknown significance in genes linked to chronic kidney disease. Four patients (4.4%) had truncation variants in genes with autosomal-recessive inheritance.

Through comprehensive genetic testing, 10 patients (11.1%) were identified as having pathogenic variants (Figure 2). Furthermore, 17 patients (18.9%) had VUS. In our study, allele frequencies of <0.001 and a Combined Annotation-Dependent Depletion score of 20 was used as the cutoff for determining the presence of pathogenic variants.^24^^,^^25^ Therefore, our definition of VUS already satisfies PM2 and PP3 of the American College of Medical Genetics and Genomics guidelines, and if the same amino acid variant has been reported to cause the disease, it satisfies PS1 and is considered a “likely pathogenic” variant. Clinical characteristics and details of pathogenic variants in these patients are shown in Table 2 and Supplementary Table S3. Previous studies reporting the same variants are listed in the Supplementary References. Of the 10 patients, 3 had *PKD1* pathogenic variants, 1 had *PKD1* and *COL4A4* pathogenic variants, and 1 had *COL4A4* and *COL4A3* pathogenic variants. In addition, 1 patient harbored a pathogenic variant in each of the following genes: *C3*, *CFHR5*, *NPHP1*, *GLA*, and *UMOD*. Through polymerase chain reaction for exons 1, 10, and 20 of *NPHP1*, no patients had an entire homozygous deletion of *NPHP1*. The comparison of clinical findings with and without pathogenic variants is shown in Table 1. There was no difference in the age of patients at RRT initiation between those with and without pathogenic variants in CKD-related genes.

**Table 2:** Clinical characteristics and pathogenic variants of 10 patients with genetic diseases

Patient No.	Sex	RRT initiation, age	Clinical diagnosis	Gene	RefSeq accession	Variant	Zygosity	gnomAD^a^	ToMMo38K^b^	CADD^c^	ACMG classification	Reports^d^	Genetic diagnosis
1412	M	30	Sporadic PKD	*PKD1*	NM_001009944	c.11693C>A; p.Ser3898∗	het.	None	None	52	Pathogenic	S1–S3	ADPKD
1475	M	42	Sporadic PKD	*PKD1*	NM_001009944	c.7927_7930delinsGC; p.Arg2643fs	het.	None	None	None	Likely Pathogenic	S4	ADPKD
1453	F	43	Sporadic PKD	*PKD1*	NM_001009944	c.1723-1G>A	het.	None	None	33	Pathogenic	S5	ADPKD
1401	F	32	Sporadic PKD	*PKD1* *COL4A4*	NM_001009944 NM_000092	c.11648_11660dup; p.Phe3888fs c.3418_3424del; p.Leu1140fs	het. het.	None None	None None	None None	Likely Pathogenic Likely Pathogenic	PKD1;S6 COL4A4; none	ADPKD Alport syndrome
1455	F	32	CGN	*C3*	NM_000064	c.943del; p.Arg315fs	het.	None	None	None	Likely Pathogenic	none	aHUS/C3GN
1423	F	43	CGN	*CFHR5*	NM_030787	c.1303C>T; p.Arg435∗	het.	0.000003983	0.000052	23	Likely Pathogenic	none	aHUS/C3GN
1449	F	46	CGN	*NPHP1*	NM_000272	c.2155G>T; p.Glu719∗	hom.	None	None	39	Likely Pathogenic	none	Nephronophthisis
1446	F	31	Renal disease in Pregnancy	*COL4A4* *COL4A3*	NM_000092 NM_000091	c.2084G>A; p.Gly695Asp c.424G>A; p.Gly142Ser	het. het.	None 0	0.000181 0.000194	23.5 24.3	Pathogenic Likely Pathogenic	COL4A4; S7–S10 COL4A3; none	Alport syndrome
1459	M	45	HNS	*GLA*	NM_000169	c.902G>A; p.Arg301Gln	het.	None	0.00003	28.1	Likely Pathogenic	S11–S34	Fabry disease
1433	M	32	Uric acid nephropathy	*UMOD*	NM_003361	c.336C>G; p.Cys112Trp	het.	None	None	22.1	Likely Pathogenic	none	ADTKD-*UMOD*

ACMG, The American College of Medical Genetics and Genomics; ADPKD, autosomal- dominant polycystic kidney disease; ADTKD, autosomal -dominant tubulointerstitial kidney disease; aHUS, atypical hemolytic uremic syndrome; C3GN, C3 glomerulopathy; CGN, chronic glomerulonephritis; F, female; het, heterozygous; HNS, hypertensive nephrosclerosis; hom, homozygous; M, male; PKD, polycystic kidney disease; RefSeq, reference sequence; RRT, renal replacement therapy.

a Genome Aggregation Database, v2.1.1.^26^

b Allele frequency panel of 38,000 Japanese individuals from The Tohoku Medical Megabank Organization.^27^

c Combined Annotation-Dependent Depletion phred score.^22^

d Previous reports were listed in the Supplementary References.

**Figure 3:**
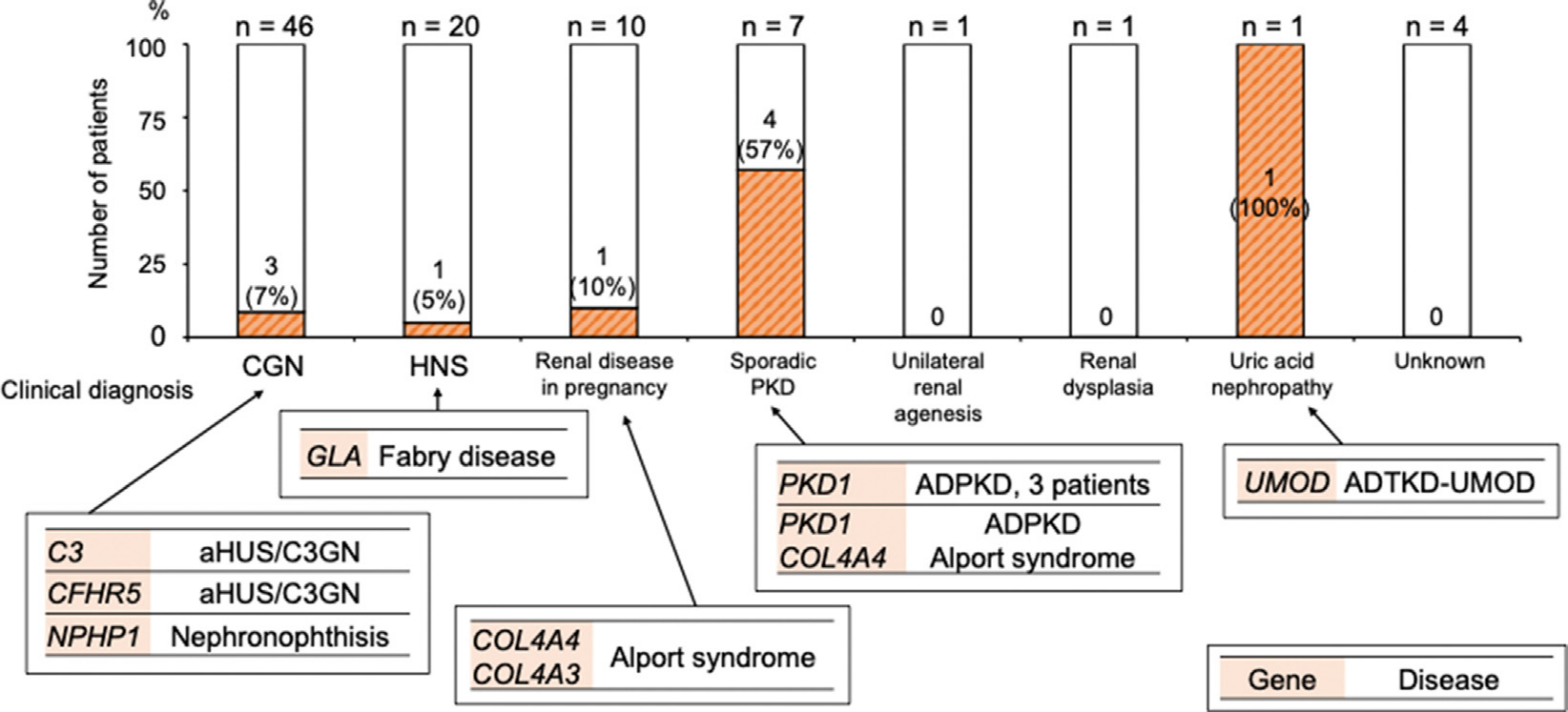
Breakdown of genetically confirmed diagnosis rates and responsible genes for each clinical diagnosis. ADPKD, autosomal -dominant polycystic kidney disease; ADTKD, autosomal-dominant tubulointerstitial kidney disease; aHUS, atypical hemolytic uremic syndrome; C3GN, C3 glomerulopathy; CGN, chronic glomerulonephritis; HNS, hypertensive nephrosclerosis; PKD, polycystic kidney disease;

**Figure 4:**
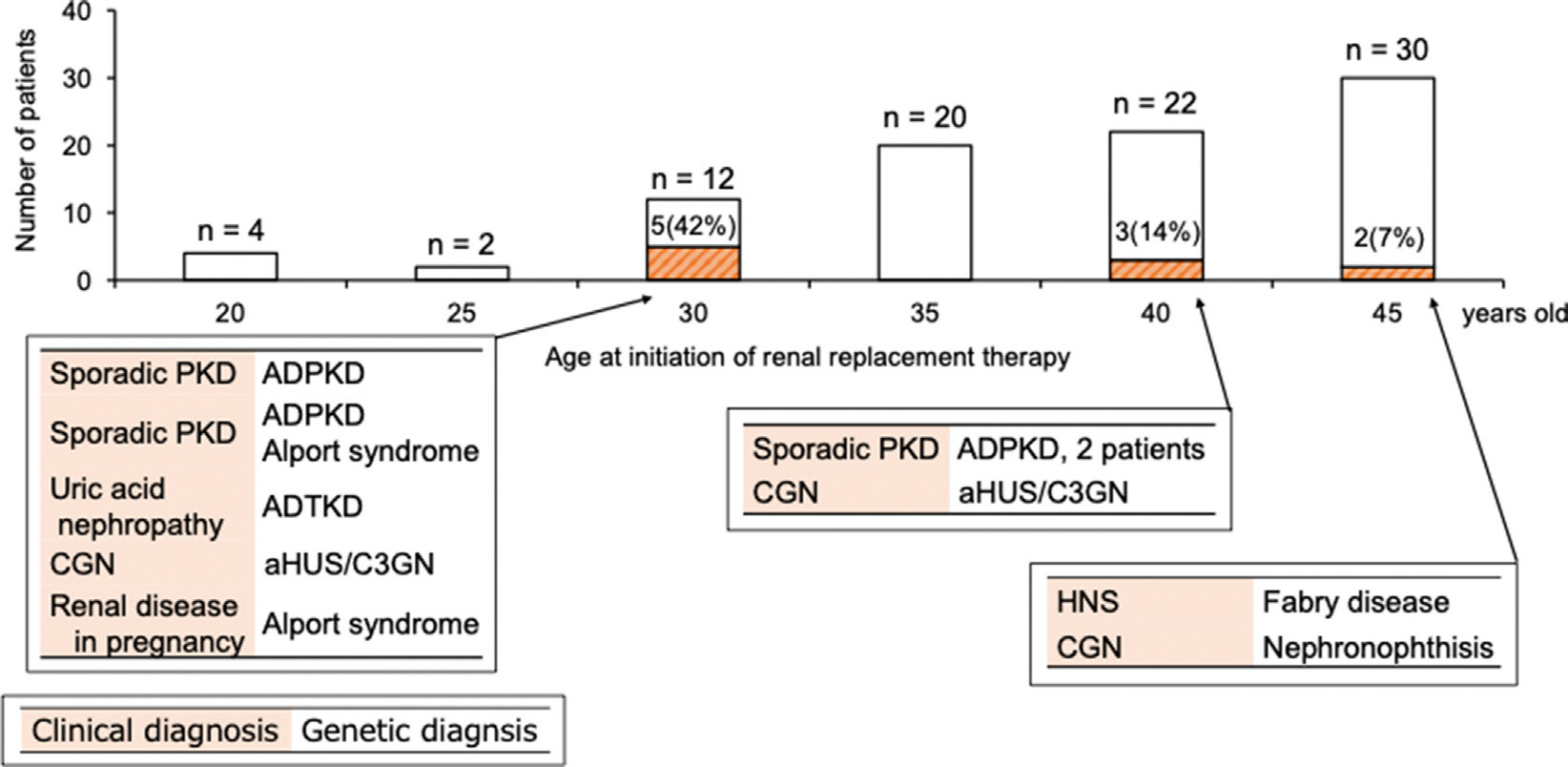
Proportions of hereditary disorders within each age group at RRT initiation. Age at RRT initiation is presented in 5-year intervals. There was no discernible correlation between the age at RRT initiation and the prevalence of genetic diseases. ADPKD, autosomal -dominant polycystic kidney disease; ADTKD, autosomal-dominant tubulointerstitial kidney disease; aHUS, atypical hemolytic uremic syndrome; C3GN, C3 glomerulopathy; CGN, chronic glomerulonephritis; PKD, polycystic kidney disease; RRT, renal replacement therapy

In Figure 3, we show the genetically confirmed diagnosis rates and the responsible genes for each clinical diagnosis. Of the 46 patients with CGN, 2 and 1 were genetically diagnosed with aHUS or C3 glomerulopathy and nephronophthisis, respectively. Of the 20 patients with hypertensive nephrosclerosis, 1 was genetically diagnosed with Fabry disease. Of the 10 patients with renal disease in pregnancy, 1 was genetically diagnosed with Alport syndrome. Of the 7 patients with sporadic PKD, 4 (57%) were genetically diagnosed with ADPKD. The patient with uric acid nephropathy was genetically diagnosed with ADTKD-*UMOD*. Four patients with an unknown diagnosis had no disease-causing variants. In Table 3, we show the comparison of the genetic diagnoses with the clinical diagnoses of patients with pathogenic variants. Four patients who were genetically diagnosed with ADPKD had concordant clinical and genetic diagnoses. However, the clinical diagnoses of patients genetically diagnosed with ADTKD, aHUS or C3 glomerulopathy, Alport syndrome, Fabry disease, and nephronophthisis were different from the genetic diagnoses. In Figure 4, we show the proportion of genetic diseases within each age group at RRT initiation. We divided the age at RRT initiation into 5-year intervals; there was no discernible correlation between the age at RRT initiation and prevalence of genetic diseases.

**Table 3:** Comparison of genetic diagnosis with clinical diagnosis

Genetic diagnosis	Clinical diagnosis					Concordance rates
	CGN	HNS	Renal disease in pregnancy	Sporadic PKD	Uric acid nephropathy	
ADPKD				3		100%
ADPKD/Alport syndrome				1		100%
ADTKD					1	0%
aHUS/C3GN	2					0%
Alport syndrome			1			0%
Fabry disease		1				0%
Nephronophthisis	1					0%

ADPKD, autosomal dominant polycystic kidney disease; ADTKD, autosomal -dominant tubulointerstitial kidney disease; aHUS, atypical hemolytic uremic syndrome; C3GN, C3 glomerulopathy; CGN, chronic glomerulonephritis; HNS, hypertensive nephrosclerosis; PKD, polycystic kidney disease.

The numbers in the cells represent the number of patients.

The medical history of patients with pathogenic variants is shown in Supplementary Table S4, and family trees of patients with pathogenic variants are shown in Supplementary Figure S1. A woman with pathogenic variants in both *PKD1* and *COL4A4* started RRT therapy at 32 years old (Patient No. 1401); she was diagnosed with PKD at 20 years old. Interestingly, her younger sister has ESRD due to nephrotic syndrome. The results of her sister’s tissue diagnosis are unknown; however, the possibility of focal segmental glomerulosclerosis due to a *COL4A4* pathogenic variant cannot be ruled out. A woman with an *NPHP1* pathogenic variant initiated RRT at 46 years old (Patient No. 1449). At 45 years old, she was noted to have liver dysfunction. Retrospectively, the possibility of extrarenal complications caused by an *NPHP1* pathogenic variant cannot be ruled out. A woman with both *COL4A3* and *COL4A4* pathogenic variants (Patient No. 1446) was diagnosed with preeclampsia while pregnant with her first child at 24 years old and CGN at 29 years old; RRT was initiated at 31 years old. A man with a *UMOD* pathogenic variant (Patient No. 1433) was diagnosed with hyperuricemia at 15 years old and uric acid nephropathy at 18 years old; he initiated RRT at 32 years old. Interestingly, his father is also maintained on dialysis due to uric acid nephropathy. Unfortunately, we were unable to perform genetic analysis on his father; however, it is highly likely that his father also has ADTKD-*UMOD*.

In addition, we detected heterozygous variants, which were categorized as VUS, in genes with autosomal dominant inheritance in 20 patients (Table 4 and Supplementary Table S5). Previous studies reporting the same variants are listed in the Supplementary References. Of the 20 patients, 3 had other pathogenic variants, whereas 17 had VUS variants only. Furthermore, in 4 patients, we detected heterozygous truncating variants in genes with AR inheritance (Supplementary Table S6). The medical history of patients with VUS in genes of autosomal dominant inheritance and truncating variants in genes with AR inheritance are shown in Supplementary Tables S7 and S8, respectively.

**Table 4:** Variants of unknown significance in genes with autosomal-dominant inheritance

Patient No.	Sex	Age on RRT	Clinical diagnosis	Gene	NCBI	Variant	GnomAD^a^	ToMMo38K^b^	CADD^c^	Report^d^	Genetic diagnosis
1398	M	38	CGN	*IFT140*	NM_014714	c.2944C>T;p.R982W	0.00003595	None	25.8	none	ADPKD
1401^e^	F	32	Sporadic PKD	*FN1*	NM_212482	c.4109G>T;p.G1370V	0.00000398	None	25.5	none	Glomeropathy with fibronectin deposits
1402	M	45	CGN	*ARHGAP24* *WT1*	NM_001025616 NM_024426	c.1448G>A;p.R483H c.698C>T;p.S233L	0.0000637 0.000003986	0.000232 None	26.5 33	none none	FSGS WT1 nephropathy
1403	M	39	CGN	*ARHGAP24* *ARHGAP24*	NM_001025616 NM_001025616	c.362G>A;p.R121H c.2087T>A;p.M969K	0.00002829 0.00001989	0.000026 None	34 29.2	none none	FSGS FSGS
1406	F	46	CGN	*APOA1*	NM_000039	c.41C>T;p.T14M	0.00002263	0.000090	24	none	Amyloidosis
1414	M	37	HNS	*FBN1*	NM_000138	c.5645C>A;p.T1882K	None	None	25.7	none	Marfan syndrome
1417	M	46	GGN	*PKD2*	NM_000297	c.149G>A;p.R50Q	0.00002029	None	23.2	none	ADPKD
1427	M	43	CGN	*FGFR1*	NM_023110	c.401C>A;p.S134Y	0.000008094	0.000258	28.1	none	Kallmann syndrome
1429	F	47	Unilateral renal agenesis	*MYH9*	NM_002473	c.4646A>T;p.Q1549L	None	0.000517	30	none	Macrothrombocytopenia and granulocyte inclusions with or without nephritis
1431	F	40	CGN	*PROKR2*	NM_144773	c.806G>A;p.C269Y	None	None	23.7	none	Kallmann syndrome
1432	M	46	CGN	*PKD2*	NM_000297	c.2005T>C;p.F669L	0.000003994	0.000439	24.3	none	ADPKD
1433 e	M	32	Uric acid nephropathy	*TSC1*	NM_000368	c.3184C>T;p.R1062W	0.0001556	None	29.9	S35–S37	TSC
1440	F	41	HNS	*TSC1*	NM_000368	c.43G>A;p.D15N	None	0.000852	33	S38	TSC
1442	M	49	Unknown	*LRP5*	NM_002335	c.2872C>T;p.R958W	0.00001592	0.000039	31	S39^,^S40	ADPLD
1449 e	F	46	CGN	*UMOD* *PKD1*	NM_003361 NM_001009944	c.1661G>A;p.R554Q c.12530C>T;p.P4177L	0.00003978 0.00002497	0.000077 0.000039	27.3 20.4	none none	ADTKD-*UMOD* ADPKD
1456	F	44	HNS	*IFT140*	NM_014714.3	c.2636A>G;p.Y879C	None	0.000052	23.6	none	ADPKD
1464	M	37	CGN	*FN1*	NM_212482	c.4858G>A;p.A1620T	0.0001631	0.000052	26.8	none	Glomeropathy with fibronectin deposits
1473	M	45	CGN	*PROK2*	NM_001126128	c.364C>T;p.R122∗	0.000003977	0.000039	44	none	Kallmann syndrome
1481	M	34	CGN	*TSC2*	NM_000548	c.3364C>T;p.R1122C	0.00006361	0.000723	25.9	none	TSC
1484	M	43	HNS	*SIX5*	NM_175875	c.947C>G;p.P316R	0.00003034	None	22.6	none	BOR syndrome

ADPKD, autosomal dominant polycystic kidney disease; ADPLD, autosomal-dominant polycystic liver disease; ADTKD, autosomal dominant tubulointerstitial kidney disease; BOR, branchio-oto-renal; CGN, chronic glomerulonephritis; F, female; FSGS, focal segmental glomerulosclerosis; HNS, hypertensive nephrosclerosis; M, male; NCBI, NCBI Reference Sequence (RefSeq) accession numbers; PKD, polycystic kidney disease; RRT, renal replacement therapy; TSC, tuberous sclerosis complex.

All variants were detected as heterozygous. All variants were classified variant of unknown significance from the American College of Medical Genetics and Genomics (ACMG) criteria.^21^

a Genome Aggregation Database, v2.1.1.^26^

b Allele frequency panel of 38,000 Japanese individuals from The Tohoku Medical Megabank Organization.^27^

c Combined Annotation-Dependent Depletion phred score.^22^

d Previous reports were listed in the Supplementary References.

e Patient No. 1401, 1033, and 1449 also had pathogenic variants (see Table 1).

## Discussion

In this study, we conducted a comprehensive genetic analysis of patients with CKD who initiated RRT between the ages of 20 and 49 and had no definitive cause of CKD. Overall, 90 patients underwent genetic testing, and 10 (11.1%) were discovered to have pathogenic variants in CKD-related genes. There was no difference in the age at RRT initiation between patients with and without pathogenic variants. Although patients who were genetically diagnosed with ADPKD had concordant clinical and genetic diagnoses, the other patients had discordant clinical and genetic diagnoses.

Compared with a previous study, ours has several strengths. First, we performed genetic analysis on adult patients on hemodialysis. Kidney transplant recipients generally have fewer comorbidities and are healthier than patients on hemodialysis.^28^^,^^29^ In addition, the distribution of CKD causes differed between patients who are eligible for and those who are not eligible for kidney transplantation.^28^ Furthermore, the percentage of patients on hemodialysis awaiting kidney transplantation is only 4% in Japan and 16% in the United States. Therefore, unlike studies on patients awaiting kidney transplantation, ours more accurately reflects the distribution of inherited kidney diseases in adult ESRD. Second, among genetic analysis studies that targeted adults with ESRD, ours included a relatively large number of patients. A genetic study on patients on a kidney transplant waitlist included those who were younger than 20 years.^16^ In addition, our cohort is approximately twice as large as that study. Although there is a large genetic study that included 1128 patients on hemodialysis, it lacked clinical information; the age at dialysis initiation was unknown, information on family history could not be collected, and genetic diagnosis was unknown.^8^ In our study, excluding sporadic cases of PKD, 6 of 83 patients (7%) had pathogenic variants. Interestingly, in a study on patients awaiting kidney transplantation, all genes with pathogenic variants were associated with glomerular disease.^16^ We genetically diagnosed not only glomerular diseases but also renal tubular diseases such as ADTKD and nephronophthisis. Therefore, our study more accurately represents the prevalence of hereditary kidney disease in adults with ESRD.

In our study, 1 patient who was clinically diagnosed as having renal disease in pregnancy had pathogenic variants in *COL4A3* and *COL4A4*. Pathogenic mutations in *COL4A3*, *COL4A4*, and *COL4A5* interfere with the synthesis and/or formation of collagen IV alpha-3-4-5 protomers and networks, resulting in a primary basement membrane disease called Alport syndrome.^30^ Heterozygous variants in *COL4A3* or *COL4A4* cause autosomal dominant Alport syndrome.^31^ In a large genetic analysis that included patients with CKD, 92 of 307 (30%) patients had *COL4A3/4/5* variants.^8^ In another study that investigated the distribution of genetic variants in adult patients with primary focal segmental glomerulosclerosis, *COL4A3/4/5* variants were the most frequent.^32^ Advances in genetics have revealed the importance of *COL4A3/4/5* variants in adults with CKD. Regarding Alport syndrome and pregnancy, a patient diagnosed with Alport syndrome with proteinuria and hematuria during pregnancy has been reported.^33^ Furthermore, some patients with Alport syndrome developed renal disorder during pregnancy that subsequently progressed to ESRD.^34^^,^^35^ Thus, the presence of genetic variants in *COL4A3/4/5* should be considered in pregnant women presenting with hematuria, proteinuria, or renal dysfunction.

In our study, 1 patient who was clinically diagnosed as having hypertensive nephrosclerosis had a pathogenic variant in *GLA*, which encodes alpha-galactosidase A. Fabry disease, which is an X-linked congenital disorder of glycosphingolipid catabolism, is caused by an alpha-galactosidase A deficiency or a deficiency in its activity.^36^ Progressive cellular accumulation of glycolipids ultimately leads to organ failure, including in the kidney.^37^ Although Fabry nephropathy manifests initially with proteinuria in the second to third decades of life,^37^ 30% to 50% of patients had hypertension from cohorts in studies regarding the natural history of Fabry disease.^38^^,^^39^ Enzyme replacement therapy or chaperone therapy, if introduced in the early stages of CKD, may prevent renal function decline and reduce proteinuria.^11^ A report on the screening of Fabry disease in 933 Japanese patients on maintenance dialysis revealed a prevalence of 0.32%.^40^ Although this rate may not be considered high even among patients with ESRD, it is crucial to note that Fabry disease is a treatable condition and should be considered as a potential differential diagnosis.

In our study, 17 (18.9%) patients had VUS in genes related to CKD. We extracted only variants that were not registered in the database of healthy individuals or that were extremely rare and had very high predictive scores for pathological significance. Although the interpretation of VUS is currently unknown, some of these variants may be responsible for CKD. Moreover, 4 patients had heterozygous truncation variants in genes with an AR inheritance. Generally, these patients are regarded as carriers and are unlikely to develop the disease. However, recent research has revealed that, although *IFT140* was initially identified as a causative gene for the AR disorder Mainzer–Saldino syndrome, monoallelic variants in *IFT140* are responsible for ADPKD.^41^^,^^42^ Therefore, the pathological significance of heterozygous variants should be carefully considered.

This study has some limitations. First, although our gene panel covered most genes associated with CKD, we did not include genes that were recently identified as disease-causing. Furthermore, due to technical limitations, we could not investigate intronic variants and *MUC1*-dupC variants that cause ADTKD-*MUC1*.^43^ In addition, we performed copy number variation analysis using next-generation sequencing data but could not perform multiplex ligation-dependent amplification or array-based comparative genome hybridization. Second, our patient selection criteria may not comprehensively encompass all individuals with inherited kidney diseases. One aspect to consider is age-related factors. Previous reports have found hereditary kidney disease in patients that initiated RRT at 50 years or older.^16^ In addition, a next-generation sequencing panel study of adult patients with CKD found that 57 of 393 (14.5%) patients with CKD aged older than 65 years had inherited kidney disease.^44^ Another consideration is the incomplete exclusion of hereditary kidney diseases from histological diagnoses. Some patients diagnosed with focal segmental glomerulosclerosis on renal biopsy also had hereditary kidney disease.^16^^,^^32^ However, these limitations only lead to an underestimation of the prevalence of hereditary kidney disease in patients who develop ESRD in adulthood.

In conclusion, our study suggests that at least 11% of patients without a definitive cause of CKD who developed kidney failure in adulthood have hereditary kidney disease. Furthermore, except for ADPKD, several inherited kidney diseases can be misdiagnosed in adult patients with ESRD. For appropriate patient management, proactive genetic analysis is needed for adult patients without a definitive cause of CKD. The pathognomonic interpretation of VUS remains inadequate, and true hereditary kidney disease may underlie many more cases.

## Disclosure

All the authors declared no conflicting interests.

## Supplementary Material

**Supplementary Methods**

**Supplementary Reference**

**Figure S1.** Family trees of patients with pathogenic variants.

**Table S1.** Targeted genes.

**Table S2.** Primer sequences of *NPHP1*.

**Table S3.** Pathogenic variants of 10 patients with genetic diseases including other scores that predicted pathogenicity.

**Table S4.** Medical history of patients with pathogenic variants.

**Table S5.** Variants of unknown significance in genes with autosomal-dominant inheritance, including other scores that predicted pathogenicity.

**Table S6.** Truncating variant in genes with an autosomal-recessive inheritance.

**Table S7.** Medical history of patients with variants of unknown significance in genes with an autosomal-dominant inheritance.

**Table S8.** Medical history of patients with truncating variants in genes with an autosomal-recessive inheritance.
