# Supplementary material for: Genetic Diagnosis of Adult Hemodialysis Patients With Unknown Etiology
Source: Kidney Int Rep. 2024 Feb 14;9(4):994–1004. doi: 10.1016/j.ekir.2024.01.027 (PMC11101786; doi:10.1016/j.ekir.2024.01.027)
Supplement: Supplementary File (PDF) [file mmc1.pdf]

Supplementary Methods

Capture-based Sequencing

RNA capture probes for the coding regions of the 298 genes were designed using Agilent's SureDesign service (https://earray.chem.agilent.com/suredesign/home.htm). Pooled and barcoded libraries for next-generation sequencing were prepared using SureSelect QXT kits (Agilent Technologies, Santa Clara, California), according to the manufacturer's protocol. Prepared libraries were sequenced by 150-bp SE reads using Illumina MiSeq sequencers. Filtering of candidate variants was achieved, as described previously[S41]. We first excluded single nucleotide variants (SNVs) with allele frequencies of >0.01 in any population within the gnomAD database[S42] (https://gnomad.broadinstitute.org/gene); National Heart, Lung, and Blood Institute Exome Sequencing Project exome variant server dataset ESP6500 (http://evs.gs.washington.edu/EVS/); 1000 Genomes catalog[S43] (http://browser.1000genomes.org/index.html); Human Genetic Variation Database[S44] (HGVD: http://www.genome. med.kyoto-u.ac.jp/SnpDB/); or the allele frequency panel of 38,000 Japanese individuals from The Tohoku Medical Megabank Organization[S45] (https://jmorp.megabank.tohoku.ac.jp). We excluded SNVs with "LOW" impact severities based on the definition in the GEMINI software[S46]; these included the following functional predictions: "synonymous_coding," "intergenic," "upstream," "UTR," "intron," etc. To interpret the significance of the variants, we used Polyphen2[S47], SIFT[S48], CADD[S49], MCAP[S50], GERP conservation scores[S51], and REVEL[S52]. The literature that was available in the Human Gene Mutation Database Pro[S53] was reviewed to assess the current evidence on the pathogenicity of previously reported variants. The methods used here were described in detail in our previous reports[S54, S55].

Supplementary References

S1. Li L. Novel human pathological mutations. Gene symbol: PKD1. Disease: human autosomal dominant polycystic kidney disease. *Hum Genet*. 2007;122(3-4):413-414.

S2. Xiong HY, Alipanahi B, Lee LJ, et al. RNA splicing. The human splicing code reveals new insights into the genetic determinants of disease. *Science*. 2015;347(6218):1254806. doi:10.1126/science.1254806

S3. Jayasinghe K, Stark Z, Kerr PG, et al. Clinical impact of genomic testing in patients with suspected monogenic kidney disease. *Genet Med*. 2021;23(1):183-191. doi:10.1038/s41436-020-00963-4

S4. Kurashige M, Hanaoka K, Imamura M, et al. A comprehensive search for mutations in the PKD1 and PKD2 in Japanese subjects with autosomal dominant polycystic kidney disease. *Clin Genet*. 2015;87(3):266-272. doi:10.1111/cge.12372

S5. Truscott L, Gell J, Chang VY, et al. Novel association of familial testicular germ cell tumor and autosomal dominant polycystic kidney disease with PKD1 mutation. *Pediatr Blood Cancer*. 2017;64(1):100-102. doi:10.1002/pbc.26197

S6. Audrézet MP, Cornec-Le Gall E, Chen JM, et al. Autosomal dominant polycystic kidney disease: comprehensive mutation analysis of PKD1 and PKD2 in 700 unrelated patients. *Hum Mutat*. 2012;33(8):1239-1250. doi:10.1002/humu.22103

S7. Oka M, Nozu K, Kaito H, et al. Natural history of genetically proven autosomal recessive Alport syndrome. *Pediatr Nephrol*. 2014;29(9):1535-1544. doi:10.1007/s00467-014-2797-4

S8. Kamiyoshi N, Nozu K, Fu XJ, et al. Genetic, Clinical, and Pathologic Backgrounds of Patients with Autosomal Dominant Alport Syndrome. *Clin J Am Soc Nephrol*. 2016;11(8):1441-1449. doi:10.2215/CJN.01000116

S9. Horinouchi T, Yamamura T, Nagano C, et al. Heterozygous Urinary Abnormality-Causing Variants of COL4A3 and COL4A4 Affect Severity of Autosomal Recessive Alport Syndrome. *Kidney360*. 2020;1(9):936-942. doi:10.34067/KID.0000372019

S10. Oh J, Shin JI, Lee K, Lee C, Ko Y, Lee JS. Clinical application of a phenotype-based NGS panel for differential diagnosis of inherited kidney disease and beyond. *Clin Genet*. 2021;99(2):236-249. doi:10.1111/cge.13869

S11. Sakuraba H, Oshima A, Fukuhara Y, et al. Identification of point mutations in the alpha-galactosidase A gene in classical and atypical hemizygotes with Fabry disease. *Am J Hum Genet*. 1990;47(5):784-789.

S12. Mills K, Morris P, Lee P, et al. Measurement of urinary CDH and CTH by tandem mass spectrometry in patients hemizygous and heterozygous for Fabry disease. *J Inherit Metab Dis*. 2005;28(1):35-48. doi:10.1007/s10545-005-5263-4

S13. Ishii S, Chang HH, Kawasaki K, et al. Mutant alpha-galactosidase A enzymes identified in Fabry disease patients with residual enzyme activity: biochemical characterization and restoration of normal intracellular processing by 1-deoxygalactonojirimycin. *Biochem J*. 2007;406(2):285-295. doi:10.1042/BJ20070479

S14. Shin SH, Murray GJ, Kluepfel-Stahl S, et al. Screening for pharmacological chaperones in Fabry disease. *Biochem Biophys Res Commun*. 2007;359(1):168-173. doi:10.1016/j.bbrc.2007.05.082

S15. Wu X, Katz E, Della Valle MC, et al. A pharmacogenetic approach to identify mutant forms of α-galactosidase A that respond to a pharmacological chaperone for Fabry disease. *Hum Mutat*.

2011;32(8):965-977. doi:10.1002/humu.21530
S16. Ishii S. Pharmacological chaperone therapy for Fabry disease. *Proc Jpn Acad Ser B Phys Biol Sci*. 2012;88(1):18-30. doi:10.2183/pjab.88.18
S17. Lukas J, Giese AK, Markoff A, et al. Functional characterisation of alpha-galactosidase a mutations as a basis for a new classification system in fabry disease. *PLoS Genet*. 2013;9(8):e1003632. doi:10.1371/journal.pgen.1003632
S18. Saito S, Ohno K, Sakuraba H. Comparative study of structural changes caused by different substitutions at the same residue on α-galactosidase A. *PLoS One*. 2013;8(12):e84267. doi:10.1371/journal.pone.0084267
S19. Brady M, Montgomery E, Brennan P, Mohindra R, Sayer JA. Diagnosing Fabry disease--delays and difficulties within discordant siblings. *QJM*. 2015;108(7):585-590. doi:10.1093/qjmed/hct024
S20. Riera C, Lois S, Domínguez C, et al. Molecular damage in Fabry disease: characterization and prediction of alpha-galactosidase A pathological mutations. *Proteins*. 2015;83(1):91-104. doi:10.1002/prot.24708
S21. Alejandra Restrepo-Cordoba M, Campuzano O, Ripoll-Vera T, et al. Usefulness of Genetic Testing in Hypertrophic Cardiomyopathy: an Analysis Using Real-World Data. *J Cardiovasc Transl Res*. 2017;10(1):35-46. doi:10.1007/s12265-017-9730-8
S22. LaDuca H, Farwell KD, Vuong H, et al. Exome sequencing covers >98% of mutations identified on targeted next generation sequencing panels. *PLoS One*. 2017;12(2):e0170843. doi:10.1371/journal.pone.0170843
S23. Nowak A, Mechtler TP, Desnick RJ, Kasper DC. Plasma LysoGb3: A useful biomarker for the diagnosis and treatment of Fabry disease heterozygotes. *Mol Genet Metab*. 2017;120(1-2):57-61. doi:10.1016/j.ymgme.2016.10.006
S24. Duro G, Zizzo C, Cammarata G, et al. Mutations in the GLA Gene and LysoGb3: Is It Really Anderson-Fabry Disease? *Int J Mol Sci*. 2018;19(12):3726. doi:10.3390/ijms19123726
S25. Nowak A, Mechtler TP, Hornemann T, et al. Genotype, phenotype and disease severity reflected by serum LysoGb3 levels in patients with Fabry disease. *Mol Genet Metab*. 2018;123(2):148-153. doi:10.1016/j.ymgme.2017.07.002
S26. Kim WS, Kim HS, Shin J, et al. Prevalence of Fabry Disease in Korean Men with Left Ventricular Hypertrophy. *J Korean Med Sci*. 2019;34(7):e63. doi:10.3346/jkms.2019.34.e63
S27. Yamamoto S, Nagasawa T, Sugimura K, et al. Clinical Diversity in Patients with Anderson-fabry Disease with the R301Q Mutation. *Intern Med*. 2019;58(4):603-607. doi:10.2169/internalmedicine.0959-18
S28. Nampoothiri S, Yesodharan D, Bhattacherjee A, et al. Fabry disease in India: A multicenter study of the clinical and mutation spectrum in 54 patients. *JIMD Rep*. 2020;56(1):82-94. doi:10.1002/jmd2.12156
S29. Mallula KK, Patel ND, Hijazi ZM, Joshi S, Naheed Z. Double-outlet right ventricle with an intact ventricular septum: a unique stage 1 palliation. *Pediatr Cardiol*. 2013;34(8):2086-2088. doi:10.1007/s00246-013-0741-1
S30. Fu L, Zhang P, Ye Q, Wu M, He L. A Case of a 49-Year-Old Man with Nonclassical Fabry Disease Diagnosed by Renal Biopsy. *Nephron*. 2022;146(1):45-48. doi:10.1159/000516924
S31. Hathaway J, Heliö K, Saarinen I, et al. Diagnostic yield of genetic testing in a heterogeneous cohort of 1376 HCM patients. *BMC Cardiovasc Disord*. 2021;21(1):126. doi:10.1186/s12872-021-01927-5
S32. Thomas DC, Sharma S, Puri RD, Verma IC, Verma J. Lysosomal storage disorders: Novel and

frequent pathogenic variants in a large cohort of Indian patients of Pompe, Fabry, Gaucher and Hurler disease. *Clin Biochem*. 2021;89:14-37. doi:10.1016/j.clinbiochem.2020.12.002
S33. Řeboun M, Sikora J, Magner M, et al. Pitfalls of X-chromosome inactivation testing in females with Fabry disease. *Am J Med Genet A*. 2022;188(7):1979-1989. doi:10.1002/ajmg.a.62728
S34. Savostyanov K, Pushkov A, Zhanin I, et al. The prevalence of Fabry disease among 1009 unrelated patients with hypertrophic cardiomyopathy: a Russian nationwide screening program using NGS technology. *Orphanet J Rare Dis*. 2022;17(1):199. doi:10.1186/s13023-022-02319-4
S35. Hoogeveen-Westerveld M, Wentink M, van den Heuvel D, et al. Functional assessment of variants in the TSC1 and TSC2 genes identified in individuals with Tuberous Sclerosis Complex. *Hum Mutat*. 2011;32(4):424-435. doi:10.1002/humu.21451
S36. Kars ME, Başak AN, Onat OE, et al. The genetic structure of the Turkish population reveals high levels of variation and admixture. *Proc Natl Acad Sci U S A*. 2021;118(36):e2026076118. doi:10.1073/pnas.2026076118
S37. Qin W, Kozlowski P, Taillon BE, et al. Ultra deep sequencing detects a low rate of mosaic mutations in tuberous sclerosis complex. *Hum Genet*. 2010;127(5):573-582. doi:10.1007/s00439-010-0801-z
S38. Togi S, Ura H, Hatanaka H, Niida Y. Genotype and Phenotype Landscape of 283 Japanese Patients with Tuberous Sclerosis Complex. *Int J Mol Sci*. 2022;23(19):11175. doi:10.3390/ijms231911175
S39. Tian T, Chen C, Zhang X, Zhang Q, Zhao P. Clinical and Genetic Features of Familial Exudative Vitreoretinopathy With Only-Unilateral Abnormalities in a Chinese Cohort. *JAMA Ophthalmol*. 2019;137(9):1054-1058. doi:10.1001/jamaophthalmol.2019.1493
S40. Li JK, Li Y, Zhang X, et al. Spectrum of Variants in 389 Chinese Probands With Familial Exudative Vitreoretinopathy. *Invest Ophthalmol Vis Sci*. 2018;59(13):5368-5381. doi:10.1167/iovs.17-23541
S41. Chong JX, Burrage LC, Beck AE, et al. Autosomal-Dominant Multiple Pterygium Syndrome Is Caused by Mutations in MYH3. *Am J Hum Genet*. 2015;96(5):841-849. doi:10.1016/j.ajhg.2015.04.004
S42. Karczewski KJ, Francioli LC, Tiao G, et al. The mutational constraint spectrum quantified from variation in 141,456 humans. *Nature*. 2020;581(7809):434-443. doi:10.1038/s41586-020-2308-7
S43. 1000 Genomes Project Consortium, Auton A, Brooks LD, et al. A global reference for human genetic variation. *Nature*. 2015;526(7571):68-74. doi:10.1038/nature15393
S44. Higasa K, Miyake N, Yoshimura J, et al. Human genetic variation database, a reference database of genetic variations in the Japanese population. *J Hum Genet*. 2016;61(6):547-553. doi:10.1038/jhg.2016.12
S45. Tadaka S, Hishinuma E, Komaki S, et al. jMorp updates in 2020: large enhancement of multi-omics data resources on the general Japanese population. *Nucleic Acids Res*. 2021;49(D1):D536-D544. doi:10.1093/nar/gkaa1034
S46. Paila U, Chapman BA, Kirchner R, Quinlan AR. GEMINI: integrative exploration of genetic variation and genome annotations. *PLoS Comput Biol*. 2013;9(7):e1003153. doi:10.1371/journal.pcbi.1003153
S47. Adzhubei IA, Schmidt S, Peshkin L, et al. A method and server for predicting damaging missense mutations. *Nat Methods*. 2010;7(4):248-249. doi:10.1038/nmeth0410-248
S48. Kumar P, Henikoff S, Ng PC. Predicting the effects of coding non-synonymous variants on protein function using the SIFT algorithm. *Nat Protoc*. 2009;4(7):1073-1081. doi:10.1038/nprot.2009.86
S49. Kircher M, Witten DM, Jain P, O'Roak BJ, Cooper GM, Shendure J. A general framework for

estimating the relative pathogenicity of human genetic variants. *Nat Genet*. 2014;46(3):310-315. doi:10.1038/ng.2892

S50. Jagadeesh KA, Wenger AM, Berger MJ, et al. M-CAP eliminates a majority of variants of uncertain significance in clinical exomes at high sensitivity. *Nat Genet*. 2016;48(12):1581-1586. doi:10.1038/ng.3703

S51. Davydov EV, Goode DL, Sirota M, Cooper GM, Sidow A, Batzoglou S. Identifying a high fraction of the human genome to be under selective constraint using GERP++. *PLoS Comput Biol*. 2010;6(12):e1001025. doi:10.1371/journal.pcbi.1001025

S52. Ioannidis NM, Rothstein JH, Pejaver V, et al. REVEL: An Ensemble Method for Predicting the Pathogenicity of Rare Missense Variants. *Am J Hum Genet*. 2016;99(4):877-885. doi:10.1016/j.ajhg.2016.08.016

S53. Stenson PD, Mort M, Ball EV, et al. The Human Gene Mutation Database: towards a comprehensive repository of inherited mutation data for medical research, genetic diagnosis and next-generation sequencing studies. *Hum Genet*. 2017;136(6):665-677. doi:10.1007/s00439-017-1779-6

S54. Fujimaru T, Mori T, Sekine A, et al. Kidney enlargement and multiple liver cyst formation implicate mutations in PKD1/2 in adult sporadic polycystic kidney disease. *Clin Genet*. 2018;94(1):125-131. doi:10.1111/cge.13249

S55. Mori T, Hosomichi K, Chiga M, et al. Comprehensive genetic testing approach for major inherited kidney diseases, using next-generation sequencing with a custom panel. *Clin Exp Nephrol*. 2017;21(1):63-75. doi:10.1007/s10157-016-1252-1

S56. Richards S, Aziz N, Bale S, et al. Standards and guidelines for the interpretation of sequence variants: a joint consensus recommendation of the American College of Medical Genetics and Genomics and the Association for Molecular Pathology. *Genet Med*. 2015;17(5):405-424. doi:10.1038/gim.2015.30

Supplementary Table S1. Targeted genes.

Gene name	OMIM MIM number	Location	Phenotype
ABCG2	603756	4q22.1	Uric acid concentration, serum, QTL1
ACE	106180	17q23.3	Renal tubular dysgenesis
ACTN4	604638	19q13.2	Glomerulosclerosis, focal segmental, 1
ADAMTS13	604134	9q34.2	Thrombotic thrombocytopenic purpura, hereditary
AGL	610860	1p21.2	Glycogen storage disease IIIa<br>Glycogen storage disease IIIb
AGTR1	106165	3q24	Renal tubular dysgenesis
AGXT	604285	2q37.3	Hyperoxaluria, primary, type 1
AHI1	608894	6q23.3	Joubert syndrome 3
ALG8	608103	11q14.1	Congenital disorder of glycosylation, type Ih<br>Polycystic liver disease 3 with or without kidney cysts
ANKFY1	607927	17p13.2	Other related gene
ANKS6	615370	9q22.33	Nephronophthisis 16
ANLN	616027	7p14.2	Focal segmental glomerulosclerosis 8
ANOS1	300836	Xp22.31	Hypogonadotropic hypogonadism 1 with or without anosmia (Kallmann syndrome 1)
APOA1	107680	11q23.3	Amyloidosis, 3 or more types<br>Hypoalphalipoproteinemia, primary, 2<br>Hypoalphalipoproteinemia, primary, 2, intermediate
APOA2	107670	1q23.3	Apolipoprotein A-II deficiency
APOA5	606368	11q23.3	Hyperchylomicronemia, late-onset
APOB	107730	2p24.1	Hypercholesterolemia, familial, 2<br>Hypobetalipoproteinemia
APOC3	107720	11q23.3	Apolipoprotein C-III deficiency
APOE	107741	19q13.32	Alzheimer disease 2<br>Hyperlipoproteinemia, type III<br>Lipoprotein glomerulopathy<br>Sea-blue histiocyte disease
APOL1	603743	22q12.3	Glomerulosclerosis, focal segmental, 4, susceptibility to
APP	104760	21q21.3	Alzheimer disease 1, familial<br>Cerebral amyloid angiopathy, Dutch, Italian, Iowa, Flemish, Arctic variants
APRT	102600	16q24.3	Adenine phosphoribosyltransferase deficiency
AQP2	107777	12q13.12	Diabetes insipidus, nephrogenic, 2

ARHGAP24	610586	4q21.3-q21.3	Other related gene
ARHGDIA	601925	17q25.3	Nephrotic syndrome, type 8
ARL13B	608922	3q11.1-q11.2	Joubert syndrome 8
ARL6	608845	3q11.2	Bardet–Biedl syndrome 3<br>Retinitis pigmentosa 55
ARMC9	617612	21q37.1	Joubert syndrome 30
ASS1	603470	9q34.11	Citrullinemia
ASS1	603470	9q34.11	Citrullinemia
ATP1A1	182310	1p13.1	Charcot–Marie–Tooth disease, axonal, type 2DD<br>Hypomagnesemia, seizures, and impaired intellectual development 2
ATP2B3	300014	Xq28	Spinocerebellar ataxia, X-linked 1
ATP6V0A1	192130	17q21.2	Developmental and epileptic encephalopathy 104<br>Neurodevelopmental disorder with epilepsy and brain atrophy
ATP6V0A4	605239	7q34	Distal renal tubular acidosis 3, with or without sensorineural hearing loss
ATP6V1B1	192132	2p13.3	Distal renal tubular acidosis 2 with progressive sensorineural hearing loss
ATP7B	606882	13q14.3	Wilson disease
AVPR2	300538	Xq28	Diabetes insipidus, nephrogenic, 1<br>Nephrogenic syndrome of inappropriate antidiuresis
B9D1	614144	17p11.2	Meckel syndrome 9<br>Joubert syndrome 27
B9D2	611951	19q13.2	Meckel syndrome 10<br>Joubert syndrome 34
BBIP1	613605	10q25.2	Bardet–Biedl syndrome 18
BBS1	209901	11q13.2	Bardet–Biedl syndrome 1
BBS10	610148	12q21.2	Bardet–Biedl syndrome 10
BBS12	610683	4q27	Bardet–Biedl syndrome 12
BBS2	606151	16q13	Bardet–Biedl syndrome 2<br>Retinitis pigmentosa 74
BBS4	600374	15q24.1	Bardet–Biedl syndrome 4
BBS5	603650	2q31.1	Bardet–Biedl syndrome 5
BBS7	607590	4q27	Bardet–Biedl syndrome 7
BBS9	615986	7p14.3	Bardet–Biedl syndrome 9

BSND	606412	1p32.3	Bartter syndrome, type 4a<br>Sensorineural deafness with mild renal dysfunction
C3	120700	19p13.3	Hemolytic uremic syndrome, atypical, susceptibility to, 5<br>C3 deficiency
CA2	611492	8q21.2	Osteopetrosis, autosomal recessive 3, with renal tubular acidosis
CACNA1D	114206	3q21.1	Primary aldosteronism, seizures, and neurologic abnormalities<br>Sinoatrial node dysfunction and deafness
CACNA1H	607904	16p13.3	Hyperaldosteronism, familial, type IV
CACNA1S	114208	1q32.1	Congenital myopathy 18 due to dihydropyridine receptor defect<br>Hypokalemic periodic paralysis, type 1
CASR	601199	3q13.33-q21.1	Hyperparathyroidism, neonatal<br>Hypocalcemia, autosomal dominant<br>Hypocalcemia, autosomal dominant, with Bartter syndrome<br>Hypocalciuric hypercalcemia, type I
CC2D2A	612013	4p15.32	COACH syndrome 2<br>Joubert syndrome 9<br>Meckel syndrome 6<br>Retinitis pigmentosa 93
CCDC28B	610162	1p35.2	Bardet–Biedl syndrome 1, modifier of
CD2AP	604241	6p12.3-p12.2	Glomerulosclerosis, focal segmental, 3
CD46	120920	1q32.2	Hemolytic uremic syndrome, atypical, susceptibility to, 2
CDC73	607393	1q31.2	Hyperparathyroidism-jaw tumor syndrome<br>Hyperparathyroidism, familial primary<br>Parathyroid adenoma with cystic changes
CDKN1B	600778	12p13.1	Multiple endocrine neoplasia, type IV
CEP104	616690	1p36.32	Intellectual developmental disorder, autosomal recessive 77<br>Joubert syndrome 25
CEP120	613446	5q23.2	Joubert syndrome 31<br>Short-rib thoracic dysplasia 13 with or without polydactyly
CEP164	614848	11q23.3	Nephronophthisis 15
CEP290	610142	12q21.32	Bardet–Biedl syndrome 14<br>Joubert syndrome 5<br>Leber congenital amaurosis 10<br>Meckel syndrome 4<br>Senior–Loken syndrome 6

CEP41	610523	7q32.2	Joubert syndrome 15
CEP83	615847	12q22	Nephronophthisis 18
CFAP418	614477	8q22.1	Bardet–Biedl syndrome 21<br>Cone-rod dystrophy 16<br>Retinitis pigmentosa 64
CFB	138470	6p21.33	Complement factor B deficiency<br>Hemolytic uremic syndrome, atypical, susceptibility to, 4
CFH	134370	1q31.3	Hemolytic uremic syndrome, atypical, susceptibility to, 1<br>Basal laminar drusen<br>Complement factor H deficiency
CFHR1	134371	1q31.3	Hemolytic uremic syndrome, atypical, susceptibility to
CFHR3	605336	1q31.3	Hemolytic uremic syndrome, atypical, susceptibility to
CFHR5	608593	1q31.3	Nephropathy due to CFHR5 deficiency
CFI	217030	4q25	Hemolytic uremic syndrome, atypical, susceptibility to, 3<br>Complement factor I deficiency
CLCN2	600570	3q27.1	Hyperaldosteronism, familial, type II<br>Leukoencephalopathy with ataxia
CLCN5	300008	Xp11.23	Dent disease 1<br>Hypophosphatemic rickets<br>Nephrolithiasis, type I<br>Proteinuria, low molecular weight, with hypercalciuric nephrocalcinosis
CLCNKA	602024	1p36.13	Bartter syndrome, type 4b, digenic
CLCNKB	602023	1p36.13	Bartter syndrome, type 3<br>Bartter syndrome, type 4b, digenic
CLDN16	603959	3q28	Hypomagnesemia 3, renal
CLDN19	610036	1p34.2	Hypomagnesemia 5, renal, with ocular involvement
CNNM2	607803	10q24.32	Hypomagnesemia 6, renal<br>Hypomagnesemia, seizures, and impaired intellectual development 1
COL4A3	120070	2q36.3	Alport syndrome 2, autosomal recessive<br>Alport syndrome 3, autosomal dominant<br>Hematuria, benign familial, 2
COL4A4	120131	2q36.3	Alport syndrome 2, autosomal recessive<br>Hematuria, familial benign, 1
COL4A5	303630	Xq22.3	Alport syndrome 1, X-linked
COL5A1	120215	9q34.3	Ehlers–Danlos syndrome, classic type, 1<br>Fibromuscular dysplasia, multifocal

COL5A2	120190	2q32.2	Ehlers–Danlos syndrome, classic type, 2
COQ2	609825	4q21.23	Coenzyme Q10 deficiency, primary, 1
COQ6	614647	14q24.3	Coenzyme Q10 deficiency, primary, 6
COQ8B	615567	19q13.2	Nephrotic syndrome, type 9
CPLANE1	614571	5p13.2	Joubert syndrome 17<br>Orofaciodigital syndrome VI
CRB2	609720	9q33.3	Focal segmental glomerulosclerosis 9<br>Ventriculomegaly with cystic kidney disease
CSPP1	611654	8q13.1-q13.2	Joubert syndrome 21
CTNNB1	116806	3p22.1	Exudative vitreoretinopathy 7<br>Neurodevelopmental disorder with spastic diplegia and visual defects
CTNS	606272	17p13.2	Cystinosis, atypical nephropathic<br>Cystinosis, late-onset juvenile or adolescent nephropathic<br>Cystinosis, nephropathic<br>Cystinosis, ocular nonnephropathic
CUBN	602997	10p13	Imerslund–Grasbeck syndrome 1
CUL3	603136	2q36.2	Neurodevelopmental disorder with or without autism or seizures<br>Pseudohypoaldosteronism, type IIE
CYP11B1	610613	8q24.3	Adrenal hyperplasia, congenital, due to 11-beta-hydroxylase deficiency<br>Aldosteronism, glucocorticoid-remediable
CYP11B2	124080	8q24.3	Aldosterone to renin ratio raised<br>Hypoaldosteronism, congenital, due to CMO I deficiency<br>Hypoaldosteronism, congenital, due to CMO II deficiency
CYP27B1	609506	12q14.1	Vitamin D-dependent rickets, type I
DCDC2	605755	6p22.3	Nephronophthisis 19<br>Sclerosing cholangitis, neonatal
DGKE	601440	17q22	Hemolytic uremic syndrome, atypical, susceptibility to, 7<br>Nephrotic syndrome, type 7
DLC1	604258	8p22	Other related gene
DMP1	600980	4q22.1	Hypophosphatemic rickets, AR
DZIP1L	617570	3q22.3	Polycystic kidney disease 5
EGF	131530	4q25	Hypomagnesemia 4, renal
EHHADH	607037	3q27.2	Fanconi renotubular syndrome 3
EMP2	602334	16p13.13	Nephrotic syndrome, type 10

ENPP1	173335	6q23.2	Arterial calcification, generalized, of infancy, 1<br>Cole disease<br>Hypophosphatemic rickets, autosomal recessive, 2
EYA1	601653	8q13.3	Anterior segment anomalies with or without cataract<br>Branchiootic syndrome 1<br>Branchiootorenal syndrome 1, with or without cataracts
FAT1	600976	4q35.2	Other related gene
FBN1	134797	15q21.1	Acromicric dysplasia<br>Ectopia lentis, familial<br>Geleophysic dysplasia 2<br>Marfan lipodystrophy syndrome<br>Marfan syndrome<br>MASS syndrome<br>Stiff skin syndrome<br>Weill–Marchesani syndrome 2, dominant
FGA	134820	4q31.3	Afibrinogenemia, congenital<br>Amyloidosis, familial visceral<br>Dysfibrinogenemia, congenital<br>Hypodysfibrinogenemia, congenital
FGF23	605380	12p13.32	Hypophosphatemic rickets, autosomal dominant<br>Tumoral calcinosis, hyperphosphatemic, familial, 2
FGFR1	136350	8p11.23	Hartsfield syndrome<br>Hypogonadotropic hypogonadism 2 with or without anosmia<br>Jackson–Weiss syndrome<br>Osteoglophonic dysplasia<br>Pfeiffer syndrome<br>Trigonocephaly 1
FN1	135600	2q35	Glomerulopathy with fibronectin deposits 2<br>Spondylometaphyseal dysplasia, corner fracture type
FOXI1	601093	5q35.1	Enlarged vestibular aqueduct
FXYD2	601814	11q23.3	Hypomagnesemia 2, renal
G6PC	613742	17q21.31	Glycogen storage disease Ia
GALNT3	601756	2q24.3	Tumoral calcinosis, hyperphosphatemic, familial, 1
GANAB	104160	11q12.3	Polycystic kidney disease 3
GAPVD1	611714	9q33.3	Other related gene
GATA3	131320	10p14	Hypoparathyroidism, sensorineural deafness, and renal dysplasia

GCM2	603716	6p24.2	Hyperparathyroidism 4<br>Hypoparathyroidism, familial isolated 2
GLA	3000644	Xq22.1	Fabry disease<br>Fabry disease, cardiac variant
GLIS2	608539	16p13.3	Nephronophthisis 7
GNA11	139313	19p13.3	Hypocalcemia, autosomal dominant 2<br>Hypocalciuric hypercalcemia, type II
GNAS	139320	20q13.32	Osseous heteroplasia, progressive<br>Pseudohypoparathyroidism Ia<br>Pseudohypoparathyroidism Ib<br>Pseudohypoparathyroidism Ic<br>Pseudopseudohypoparathyroidism
GNASAS1	610540	20q13.32	Pseudohypoparathyroidism, type IB
GPIHBP1	612757	8q24.3	Hyperlipoproteinemia, type 1D
GRHPR	604296	9p13.2	Hyperoxaluria, primary, type II
GSN	137350	9q33.2	Amyloidosis, Finnish type
HNF1B	189907	17q12	Renal cysts and diabetes syndrome<br>Type 2 diabetes mellitus
HNF4A	600281	20q13.12	Fanconi renotubular syndrome 4, with maturity-onset diabetes of the young<br>MODY, type I
HOGA1	613597	10q24.2	Hyperoxaluria, primary, type III
HPRT1	308000	Xq26.2-q26.3	Hyperuricemia, HRPT-related<br>Lesch–Nyhan syndrome
HSD11B2	614232	16q22.1	Apparent mineralocorticoid excess
IFT122	606045	3q21.3-q22.1	Cranioectodermal dysplasia 1
IFT140	614620	16p13.3	Retinitis pigmentosa 80<br>Short-rib thoracic dysplasia 9 with or without polydactyly
IFT172	607386	2p23.3	Bardet–Biedl syndrome 20<br>Retinitis pigmentosa 71<br>Short-rib thoracic dysplasia 10 with or without polydactyly
IFT27	615870	22q12.3	Bardet–Biedl syndrome 19
IFT43	614068	14q24.3	Short-rib thoracic dysplasia 18 with polydactyly
IFT74	608040	9p21.2	Bardet–Biedl syndrome 22<br>Joubert syndrome 40<br>Spermatogenic failure 58

INF2	610982	14q32.33	Charcot–Marie–Tooth disease, dominant intermediate E<br>Glomerulosclerosis, focal segmental, 5
INPP5E	613037	9q34.3	Joubert syndrome 1<br>Mental retardation, truncal obesity, retinal dystrophy, and micropenis
INVS	243305	9q31.1	Nephronophthisis 2, infantile
IQCB1	609237	3q13.33	Senior–Loken syndrome 5
ITGA3	605025	17q21.33	Epidermolysis bullosa, junctional 7, with interstitial lung disease and nephrotic syndrome
ITGB4	147557	17q25.1	Epidermolysis bullosa, junctional 5A, intermediate<br>Epidermolysis bullosa, junctional 5B, with pyloric atresia
ITSN1	602442	21q22.11	Other related gene
ITSN2	604464	2p23.3	Other related gene
KATNIP	616650	16p12.1	Joubert syndrome 26
KCNA1	176260	12p13.32	Episodic ataxia/myokymia syndrome
KCNJ1	600359	11q24.3	Bartter syndrome, type 2
KCNJ10	602208	1q23.2	Enlarged vestibular aqueduct, digenic<br>SESAME syndrome
KCNJ5	600734	11q24.3	Hyperaldosteronism, familial, type III<br>Long QT syndrome 13
KIAA0586	610178	14q23.1	Joubert syndrome 23<br>Short-rib thoracic dysplasia 14 with polydactyly
KIF14	611279	1q32.1	Meckel syndrome 12<br>Microcephaly 20, primary, autosomal recessive
KIF7	611254	15q26.1	Acrocallosal syndrome<br>Joubert syndrome 12
KL	604824	13q13.1	Tumoral calcinosis, hyperphosphatemic, familial, 3
KLHL2	605774	4q32.3	Other related gene
KLHL3	605775	5q31.2	Pseudohypoaldosteronism, type IID
LAMB2	150325	3p21.31	Nephrotic syndrome, type 5, with or without ocular abnormalities<br>Pierson syndrome
LDLR	606945	19p13.2	Hypercholesterolemia, familial, 1<br>LDL cholesterol level QTL2
LMX1B	602575	9q33.3	Focal segmental glomerulosclerosis 10<br>Nail–patella syndrome
LPL	609708	8p21.3	Combined hyperlipidemia, familial<br>Lipoprotein lipase deficiency

LRBA	606453	4q31.3	Immunodeficiency, common variable, 8, with autoimmunity
LRP5	603506	11q13.2	Endosteal hyperostosis<br>Exudative vitreoretinopathy 4<br>Osteopetrosis, autosomal dominant 1<br>Osteoporosis-pseudoglioma syndrome<br>Polycystic liver disease 4 with or without kidney cysts
LYZ	153450	12q15	Amyloidosis, renal
MAGED2	300470	Xp11.21	Bartter syndrome, type 5, antenatal, transient
MAGI2	606382	7q21.11	Nephrotic syndrome, type 15
MAPKBP1	616786	15q15.1	Nephronophthisis 20
MEFV	608107	16p13.3	Familial Mediterranean fever, AD<br>Familial Mediterranean fever, AR<br>Neutrophilic dermatosis, acute febrile
MEN1	613733	11q13.1	Multiple endocrine neoplasia 1
MKKS	604896	20p12.2	Bardet–Biedl syndrome 6<br>McKusick–Kaufman syndrome
MKS1	609883	17q22	Bardet–Biedl syndrome 13<br>Joubert syndrome 28<br>Meckel syndrome 1
MUC1	158340	1q22	Tubulointerstitial kidney disease, autosomal dominant, 2
MYH9	160775	22q12.3	Deafness, autosomal dominant 17<br>Macrothrombocytopenia and granulocyte inclusions with or without nephritis or sensorineural hearing loss
MYO1E	601479	15q22.2	Glomerulosclerosis, focal segmental, 6
NEDD4L	606384	18q21.31	Periventricular nodular heterotopia 7
NEK8	609799	17q11.2	Nephronophthisis 9<br>Renal-hepatic-pancreatic dysplasia 2
NLRP3	606416	1q44	CINCA syndrome<br>Deafness, autosomal dominant 34, with or without inflammation<br>Familial cold inflammatory syndrome 1<br>Keratoendothelitis fugax hereditaria<br>Muckle–Wells syndrome
NOTCH2	600275	1p12	Alagille syndrome 2<br>Hajdu–Cheney syndrome

NPHP1	607100	2q13	Joubert syndrome 4<br>Nephronophthisis 1, juvenile<br>Senior–Loken syndrome-1
NPHP3	608002	3q22.1	Meckel syndrome 7<br>Nephronophthisis 3<br>Renal-hepatic-pancreatic dysplasia 1
NPHP4	607215	1p36.31	Nephronophthisis 4<br>Senior–Loken syndrome 4
NPHS1	602716	19q13.12	Nephrotic syndrome, type 1
NPHS2	604766	1q25.2	Nephrotic syndrome, type 2
NR3C2	600983	4q31.23	Hypertension, early-onset, autosomal dominant, with exacerbation in pregnancy<br>Pseudohypoaldosteronism type I, autosomal dominant
NUP107	607617	12q15	Galloway–Mowat syndrome 7<br>Nephrotic syndrome, type 11
NUP133	607613	1q42.13	Galloway–Mowat syndrome 8<br>Nephrotic syndrome, type 18
NUP160	607614	11p11.2	Nephrotic syndrome, type 19
NUP205	614352	7q33	Nephrotic syndrome, type 13
NUP85	170285	17q25.1	Nephrotic syndrome, type 17
NUP93	614351	16q13	Nephrotic syndrome, type 12
OCRL	300535	Xq26.1	Dent disease 2<br>Lowe syndrome
OFD1	300170	Xp22.2	Joubert syndrome 10<br>Orofaciodigital syndrome I<br>Simpson–Golabi–Behmel syndrome, type 2
OXSR1	604046	3p22.2	Other related gene
PAX2	167409	10q24.31	Glomerulosclerosis, focal segmental, 7<br>Papillorenal syndrome
PCBD1	126090	10q22.1	Hyperphenylalaninemia, BH4-deficient, D
PCSK9	607786	1p32.3	Hypercholesterolemia, familial, 3
PDE6D	602676	2q37.1	Joubert syndrome 22
PDSS2	610564	6q21	Coenzyme Q10 deficiency, primary, 3
PFKM	610681	12q13.11	Glycogen storage disease VII
PHEX	300550	Xp22.11	Hypophosphatemic rickets, X-linked dominant
PIBF1	607532	13q21.33-q22.1	Joubert syndrome 33

PKD1	601313	16p13.3	Polycystic kidney disease 1
PKD2	173910	4q22.1	Polycystic kidney disease 2
PKHD1	606702	6p12.3-p12.2	Polycystic kidney disease 4, with or without hepatic disease
PLCE1	608414	10q23.33	Nephrotic syndrome, type 3
PLG	173350	6q26	Angioedema, hereditary, 4<br>Dysplasminogenemia<br>Plasminogen deficiency, type I
PODXL	602632	7q32.3	Other related gene
PRKCSH	177060	19p13.2	Polycystic liver disease 1
PROK2	607002	3p13	Hypogonadotropic hypogonadism 4 with or without anosmia
PROKR2	607123	20p12.3	Hypogonadotropic hypogonadism 3 with or without anosmia
PRPS1	311850	Xq22.3	Arts syndrome<br>Charcot–Marie–Tooth disease, X-linked recessive, 5<br>Deafness, X-linked 1<br>Gout, PRPS-related<br>Phosphoribosylpyrophosphate synthetase superactivity
PTH	168450	11p15.3	Hypoparathyroidism, familial isolated 1
PTPRO	600579	12p12.3	Nephrotic syndrome, type 6
PYGM	608455	11q13.1	McArdle disease
REN	179820	1q32.1	Renal tubular dysgenesis<br>Tubulointerstitial kidney disease, autosomal dominant, 4
RET	164761	10q11.21	Medullary thyroid carcinoma<br>Multiple endocrine neoplasia IIA<br>Multiple endocrine neoplasia IIB<br>Pheochromocytoma
RPGRIP1L	610937	16q12.2	Joubert syndrome 7<br>Meckel syndrome 5
SALL1	602218	16q12.1	Townes–Brocks branchiootorenal-like syndrome<br>Townes–Brocks syndrome 1
SARS2	612804	19q13.2	Hyperuricemia, pulmonary hypertension, renal failure, and alkalosis

SCN4A	603967	17q23.3	Congenital myopathy 22A, classic<br>Congenital myopathy 22B, severe fetal<br>Hyperkalemic periodic paralysis<br>Hypokalemic periodic paralysis, type 2<br>Myasthenic syndrome, congenital, 16<br>Myotonia congenita, atypical, acetazolamide-responsive<br>Paramyotonia congenita
SCNN1A	600228	12p13.31	Liddle syndrome 3<br>Bronchiectasis with or without elevated sweat chloride 2<br>Pseudohypoaldosteronism, type IB1, autosomal recessive
SCNN1B	600760	16p12.2	Bronchiectasis with or without elevated sweat chloride 1<br>Liddle syndrome 1<br>Pseudohypoaldosteronism, type IB2, autosomal recessive
SCNN1G	600761	16p12.2	Bronchiectasis with or without elevated sweat chloride 3<br>Liddle syndrome 2<br>Pseudohypoaldosteronism, type IB3, autosomal recessive
SDCCAG8	613524	1q43-q44	Bardet–Biedl syndrome 16<br>Senior–Loken syndrome 7
SEC61A1	609213	3q21.3	Tubulointerstitial kidney disease, autosomal dominant, 5
SEC61B	609214	9q22.33	Other related gene
SEC63	608648	6q21	Polycystic liver disease 2
SGK1	602958	6q23.2	Other related gene
SIX1	601205	14q23.1	Branchiootic syndrome 3<br>Deafness, autosomal dominant 23
SIX5	600963	19q13.32	Branchiootorenal syndrome 2
SLC12A1	600839	15q21.1	Bartter syndrome, type 1
SLC12A3	600968	16q13	Gitelman syndrome
SLC16A12	611910	10q23.31	Cataract 47, juvenile, with microcornea
SLC17A5	604322	6q13	Salla disease<br>Sialic acid storage disorder, infantile
SLC22A12	607096	11q13.1	Hypouricemia, renal
SLC26A3	126650	7q22.3-q31.1	Diarrhea 1, secretory chloride, congenital
SLC26A4	605646	7q22.3	Deafness, autosomal recessive 4, with enlarged vestibular aqueduct<br>Pendred syndrome
SLC2A2	138160	3q26.2	Fanconi–Bickel syndrome
SLC2A9	606142	4p16.1	Hypouricemia, renal, 2

SLC34A1	182309	5q35.3	Fanconi renotubular syndrome 2<br>Hypercalcemia, infantile, 2<br>Nephrolithiasis/osteoporosis, hypophosphatemic, 1
SLC34A3	609826	9q34.3	Hypophosphatemic rickets with hypercalciuria
SLC37A4	602671	11q23.3	Congenital disorder of glycosylation, type IIw<br>Glycogen storage disease Ib<br>Glycogen storage disease Ic
SLC3A1	104614	2p21	Cystinuria
SLC41A1	610801	1q32.1	Nephronophthisis-like nephropathy 2
SLC4A1	109270	17q21.31	Cryohydrocytosis<br>Distal renal tubular acidosis 1<br>Distal renal tubular acidosis 4 with hemolytic anemia<br>Ovalocytosis, SA type<br>Spherocytosis, type 4
SLC4A4	603345	4q13.3	Renal tubular acidosis, proximal, with ocular abnormalities
SLC5A1	182380	22q12.3	Glucose/galactose malabsorption
SLC5A2	182381	16p11.2	Renal glucosuria
SLC7A9	604144	19q13.11	Cystinuria
SLC9A3	182307	5p15.33	Diarrhea 8, secretory sodium, congenital
SMARCAL1	606622	2q35	Schimke immunoosseous dysplasia
SQSTM1	601530	5q35.3	Frontotemporal dementia and/or amyotrophic lateral sclerosis 3<br>Myopathy, distal, with rimmed vacuoles<br>Neurodegeneration with ataxia, dystonia, and gaze palsy, childhood-onset<br>Paget disease of bone 3
STAP1	604298	4q13.2	Other related gene
STK39	607648	2q24.3	Other related gene
STX16	603666	20q13.32	Pseudohypoparathyroidism, type IB
SUFU	607035	10q24.32	Joubert syndrome 32
TBC1D8B	301027	Xq22.3	Nephrotic syndrome, type 20
TCTN1	609863	12q24.11	Joubert syndrome 13
TCTN2	613846	12q24.31	Meckel syndrome 8<br>Joubert syndrome 24
TCTN3	613847	10q24.1	Joubert syndrome 18<br>Orofaciodigital syndrome IV
THBD	188040	20p11.21	Hemolytic uremic syndrome, atypical, susceptibility to, 6<br>Thrombophilia 12 due to thrombomodulin defect

TMEM107	616183	17p13.1	Joubert syndrome 29<br>Meckel syndrome 13<br>Orofaciodigital syndrome XVI
TMEM138	614459	11q12.2	Joubert syndrome 16
TMEM216	613277	11q12.2	Joubert syndrome 2<br>Meckel syndrome 2
TMEM231	614949	16q23.1	Joubert syndrome 20<br>Meckel syndrome 11
TMEM237	614423	2q33.1	Joubert syndrome 14
TMEM67	609884	8q22.1	COACH syndrome 1<br>Joubert syndrome 6<br>Meckel syndrome 3<br>Nephronophthisis 11
TNS2	607717	12q13.13	Other related gene
TNXB	600985	6p21.33-p21.32	Ehlers–Danlos syndrome, classic-like, 1<br>Vesicoureteral reflux 8
TRAF3IP1	607380	2q37.3	Senior–Loken syndrome 9
TRIM32	602290	9q33.1	Bardet–Biedl syndrome 11<br>Muscular dystrophy, limb-girdle, autosomal recessive 8
TRPC6	603652	11q22.1	Glomerulosclerosis, focal segmental, 2
TRPM6	607009	9q21.13	Hypomagnesemia 1, intestinal
TSC1	605284	9q34.13	Tuberous sclerosis-1
TSC2	191092	16p13.3	Tuberous sclerosis-2
TTC21B	612014	2q24.3	Nephronophthisis 12<br>Short-rib thoracic dysplasia 4 with or without polydactyly
TTC21B	612014	2q24.3	Nephronophthisis 12<br>Short-rib thoracic dysplasia 4 with or without polydactyly
TTC8	608132	14q31.3	Bardet–Biedl syndrome 8
TTR	176300	18q12.1	Amyloidosis, hereditary, transthyretin-related<br>Carpal tunnel syndrome, familial
UMOD	191845	16p12.3	Tubulointerstitial kidney disease, autosomal dominant, 1
VDR	601769	12q13.11	Rickets, vitamin D-resistant, type IIA
VHL	608537	3p25.3	Erythrocytosis, familial, 2<br>Pheochromocytoma<br>von Hippel–Lindau syndrome

WDPCP	613580	2p15	Bardet–Biedl syndrome 15<br>Congenital heart defects, hamartomas of tongue, and polysyndactyly
WDR19	608151	4p14	Cranioectodermal dysplasia 4<br>Nephronophthisis 13<br>Senior–Loken syndrome 8<br>Short-rib thoracic dysplasia 5 with or without polydactyly
WDR35	613602	2p24.1	Cranioectodermal dysplasia 2<br>Short-rib thoracic dysplasia 7 with or without polydactyly
WDR72	613214	15q21.3	Amelogenesis imperfecta, type IIA3
WNK1	605232	12p13.33	Neuropathy, hereditary sensory and autonomic, type II<br>Pseudohypoaldosteronism, type IIC
WNK2	606249	9q22.31	Other related gene
WNK3	300358	Xp11.22	Other related gene
WNK4	601844	17q21.2	Pseudohypoaldosteronism, type IIB
WT1	607102	11p13	Denys–Drash syndrome<br>Frasier syndrome<br>Meacham syndrome<br>Nephrotic syndrome, type 4<br>Wilms tumor, type 1
XDH	607633	2p23.1	Xanthinuria, type I
XPNPEP3	613553	22q13.2	Nephronophthisis-like nephropathy 1
ZNF423	604557	16q12.1	Joubert syndrome 19<br>Nephronophthisis 14

OMIM, Online Mendelian Inheritance in Man.

Supplementary Table S2. Primer sequences of *NPHP1*.

Amplified Region [a]	Primer	Sequence	Product size (bp)	Annealing Temperature (ºC)
Exon 1	Forward	GCCGGTTGGTTTCCCTG	209	60
	Reverse	TAGGTGGGGTCACGGTGG		
Exon 10	Forward	GGCATTTTGGAAGTGCCTG	258	60
	Reverse	TTGCAACTATGACAAAATCTGG		
Exon 20	Forward	TCCTACCTCTTAGGTGGCTTTTAG	509	60
	Reverse	AATCGTGGAGGATCCATCTG		

[a] The following NCBI reference sequences were used: *NPHP1*, NM_000272.3

Supplementary Table S3. Pathogenic variants of 10 patients with genetic diseases including other scores that predicted pathogenicity.

Patient No.	Gene	RefSeq accession	Variant	Zygosity	Polyphen2	SIFT	MCAP	GERP	REVEL
1412	*PKD1*	NM_001009944	c.11693C>A; p.Ser3898*	het.	None	0.016	None	5.12	None
1475	*PKD1*	NM_001009944	c.7927_7930delinsGC; p.Arg2643fs	het.	None	None	None	None	None
1453	*PKD1*	NM_001009944	c.1723-1G>A	het.	None	None	None	4.86	None
1401	*PKD1*	NM_001009944	c.11648_11660dup; p.Phe3888fs	het.	None	None	None	None	None
	COL4A4	NM_000092	c.3418_3424del; p.Leu1140fs	het.	None	None	None	None	None
1455	*C3*	NM_000064	c.943del; p.Arg315fs	het.	None	0.267	None	-1.97	None
1423	*CFHR5*	NM_030787	c.1303C>T; p.Arg435*	het.	None	None	None	-7.51	None
1449	*NPHP1*	NM_000272	c.2155G>T; p.Glu719*	hom.	None	None	None	None	None
1446	*COL4A4*	NM_000092	c.2084G>A; p.Gly695Asp	het.	1	0	0.92	5.75	0.932
	COL4A3	NM_000091	c.424G>A; p.Gly142Ser	het.	1	0.004	0.864	5.49	0.947
1459	*GLA*	NM_000169	c.902G>A; p.Arg301Gln	het.	1	0.006	0.954	5.05	0.951
1433	*UMOD*	NM_003361	c.336C>G; p.Cys112Trp	het.	1	0.001	0.902	1.11	0.825

Supplementary Table S4. Medical history of patients with pathogenic variants.

Patient No.	Sex	RRT initiation age	Clinical diagnosis	Medical history	Family history	Pathogenic variants	Genetic diagnosis
1412	M	30	Sporadic PKD	Hypertension was noted at the age of 15. Diagnosed with PKD at age 29. No cerebral aneurysms.	None.	*PKD1*: p.S3898*	ADPKD
1475	M	42	Sporadic PKD	At the age of 41, he had hematuria.	Father developed ESRD due to CGN.	*PKD1*: p.R2643fs	ADPKD
1453	F	43	Sporadic PKD	Renal function worsened after preeclampsia.	None.	*PKD1*: p.1723-1G>A	ADPKD
1401	F	32	Sporadic PKD	Diagnosed with PKD at age 20. Complicated by PLD.	Sister developed ESRD due to NS. Brother had diabetes.	*PKD1*: p.F3888fs *COL4A4*: p.L1140fs	ADPKD Alport syndrome
1455	F	32	CGN	Diagnosed with CKD at age 29. Renal function worsened during pregnancy at the age of 31.	None.	*C3*: p.R315fs	aHUS/C3GN
1423	F	43	CGN	Recurrent tonsillitis in childhood. Proteinuria and hematuria were noted at the age of 20. She had pyelonephritis twice at age 25 and once at age 29.	Father had hypertension. Mother had lung cancer.	*CFHR5*: p.R435*	aHUS/C3GN

1449	F	46	CGN	At the age of 45, she was diagnosed with liver dysfunction, anemia, and CKD.	None.	*NPHP1*: p.E719*	Nephronophthisis
1446	F	31	Renal disease in Pregnancy	While pregnant with her first child at the age of 24, she suffered from preeclampsia. Proteinuria and hematuria were noted at the age of 29.	None.	*COL4A4*: p.G695D *COL4A3*: p.G142S	Alport syndrome
1459	M	45	HNS	Mental retardation. Diagnosed with hypertension and CKD at age 45. Decreased α-Gal A leukocyte activity was observed after genetic diagnosis.	Father had valvular heart disease. Mother has arterial fibrillation.	*GLA*: p.R301Q	Fabry disease
1433	M	32	Uric acid nephropathy	Hyperuricemia was noted at the age of 15. Diagnosed with uric acid nephropathy at the age of 18.	Father developed ESRD due to uric acid nephropathy.	*UMOD*: p.C112W	ADTKD-*UMOD*

M, male; F, female, RRT, renal replacement therapy; PKD, polycystic kidney disease; CGN, chronic glomerulonephritis; HNS, hypertensive nephrosclerosis; CKD, chronic kidney disease; PLD, polycystic liver disease; Gal, galactosidase; ESRD, end-stage renal disease; NS, nephrotic syndrome; ADPKD, autosomal dominant polycystic kidney disease; aHUS, atypical hemolytic uremic syndrome; C3GN, C3 glomerulopathy; ADTKD, autosomal dominant tubulointerstitial kidney disease.

Supplementary Table S5. Variants of unknown significance in genes with autosomal-dominant inheritance, including other scores that predicted pathogenicity.

Patient No.	Gene	RefSeq accession	Variant	Polyphen2	SIFT	MCAP	GERP	REVEL
1398	*IFT140*	NM_014714	c.2944C>T;p.R982W	0.976	0	0.016	5.53	0.161
1401[a]	*FN1*	NM_212482	c.4109G>T;p.G1370V	0.962	0.176	0.081	5.61	0.596
1402	*ARHGAP24*	NM_001025616	c.1448G>A;p.R483H	1	0.007	0.054	5.72	0.178
	WT1	NM_024426	c.698C>T;p.S233L	0.994	0	0.132	5.45	0.691
1403	*ARHGAP24*	NM_001025616	c.362G>A;p.R121H	0.997	0.074	0.039	6.17	0.419
	ARHGAP24	NM_001025616	c.2087T>A;p.M969K	0.987	0.007	0.054	5.56	0.422
1406	*APOA1*	NM_000039	c.41C>T;p.T14M	1	0.001	0.255	4.89	0.45
1414	*FBN1*	NM_000138	c.5645C>A;p.T1882K	0.946	0.121	0.095	6.16	0.759
1417	*PKD2*	NM_000297	c.149G>A;p.R50Q	0.003	0.106	0.69	2.88	0.054
1427	*FGFR1*	NM_023110	c.401C>A;p.S134Y	0.992	0.012	0.26	5.69	0.433
1429	*MYH9*	NM_002473	c.4646A>T;p.Q1549L	0.98	0.001	0.836	5.25	0.742
1431	*PROKR2*	NM_144773	c.806G>A;p.C269Y	0.808	0.041	0.032	5.05	0.23
1432	*PKD2*	NM_000297	c.2005T>C;p.F669L	0.25	0.026	0.226	4.28	0.528
1433 [a]	*TSC1*	NM_000368	c.3184C>T;p.R1062W	0.999	0.013	0.207	3.45	0.681
1440	*TSC1*	NM_000368	c.43G>A;p.D15N	0.976	0.032	0.119	6.17	0.395
1442	*LRP5*	NM_002335	c.2872C>T;p.R958W	1	0	0.749	4.36	0.842
1449 [a]	*UMOD*	NM_003361	c.1661G>A;p.R554Q	0.981	0.135	0.054	4.67	0.396
	PKD1	NM_001009944	c.12530C>T;p.P4177L	1	0.004	0.096	2.21	0.26
1456	*IFT140*	NM_014714.3	c.2636A>G;p.Y879C	0.999	0	0.092	2.67	0.696
1464	*FN1*	NM_212482	c.4858G>A;p.A1620T	0.999	0.105	0.017	6.03	0.327

1473	*PROK2*	NM_001126128	c.364C>T;p.R122*	None	None	None	5.86	None
1481	*TSC2*	NM_000548	c.3364C>T;p.R1122C	0.004	0.013	0.029	2.58	0.537
1484	*SIX5*	NM_175875	c.947C>G;p.P316R	0.999	0.017	0.287	4.2	0.285

a Patient No. 1401, 1033, and 1449 also had pathogenic variants (see Table 1).

Supplementary Table S6. Truncating variant in genes with autosomal-recessive inheritance.

Patient No.	Sex	Age on RRT	Clinical diagnosis	Gene	NCBI	Variant	GnomAD[a]	ToMMo38K[b]	CADD[c]	Reports	Genetic diagnosis
1424	M	39	CGN	*NPHP4*	NM_015102	c.2817+1G>A	None	None	29.5	none	NPHP<br>SLSN
1428	F	46	CGN	*IFT172*	NM_015662	c.615G>A;p.W205*	None	None	36	none	NPHP<br>BBS<br>Skeletal ciliopathy
1450	F	44	HNS	*KIAA0586*	NM_001244189	c.44del;p.G15fs	None	None	17.97	none	JBTS
1467	M	36	HNS	*WDPCP*	NM_015910	c.541_542del;p.Q181fs	None	None	29.6	none	BBS

All variants were detected as heterozygous. All variants were classified likely pathogenic variant from the American College of Medical Genetics and Genomics criteria[S56].

a Genome Aggregation Database, v2.1.1[S42].

b Allele frequency panel of 38,000 Japanese individuals from The Tohoku Medical Megabank Organization[S45].

c Combined Annotation-Dependent Depletion phred score[S49].

M, male; F, female; RRT, renal replacement therapy; CGN, chronic glomerulonephritis; HNS, hypertensive nephrosclerosis; NCBI, NCBI Reference Sequence (RefSeq) accession numbers; NPHP, nephronophthisis; SLSN, Senior Løken syndrome; BBS, Bardet–Biedl syndrome; JBTS, Joubert syndrome.

Supplementary Table S7. Medical history of patients with variant of unknown significance in genes with an autosomal-dominant inheritance.

Patient N.	Sex	RRT initiation age	Clinical diagnosis	Medical history	Family history	Variants	Genetic diagnosis
1398	M	38	CGN	Acute nephritis occurred at the age of 23. After that, proteinuria was occasionally observed.	None.	*IFT140*: p.R982W	ADPKD
1402	M	45	CGN	Hypertension was noted at the age of 28. However, he did not seek medical attention.	None.	*ARHGAP24*: p.R483H<br>*WT1*: p.S233L	FSGS<br>WT1 nephropathy
1403	M	39	CGN	Acute nephritis occurred at the age of 9. After that, proteinuria was occasionally observed.	None.	*ARHGAP24*: p.R121H<br>*ARHGAP24*: p.M969K	FSGS<br>FSGS
1406	F	46	CGN	Hypertension and proteinuria were noted at the age of 20.<br>Diagnosed with diabetes at age 45.<br>Renal function worsened at age 46.	None.	*APOA1*: p.T14M	Amyloidosis
1414	M	37	HNS	Proteinuria and hematuria were noted at age of 30.	None.	*FBN1*: p.T1882K	Marfan syndrome
1417	M	46	CGN	Hematuria was noted at age of 15. Diagnosed gout at age 32.<br>Diagnosed with hypertension and CKD at	Parents have cerebral infarction. Father has diabetes.	*PKD2*: p.R50Q	ADPKD

				age 40.			
1427	M	43	CGN	Diagnosed NS at age 12. Diagnosed CGN at age 31.	None.	*FGFR1*: p.S134Y	Kallmann syndrome
1429	F	47	Unilateral renal agenesis	Proteinuria was noted at age of 24. Diagnosed with unilateral renal agenesis at age 28.	Farther has cerebral hemorrhage. Son has hydrocephalus.	*MYH9*: p.Q1549L	Macrothrombocytopenia and granulocyte inclusions with or without nephritis
1431	F	40	CGN	Diagnosed with pyelonephritis at age 25.	None.	*PROKR2*: p.C269Y	Kallmann syndrome
1432	M	46	CGN	Hypertension was noted at the age of 32. However, he did not seek medical attention. Diagnosed with CKD at age 43.	Father has esophagial cancer.	*PKD2*: p.F669L	ADPKD
1440	F	41	HNS	Diagnosed with hypertension and CKD at age 41.	None.	*TSC1*: p.D15N	TSC
1442	M	49	Unknown	Unknown.	None.	*LRP5*: p.R958W	ADPLD
1456	F	42	CGN	Suspected CGN at age 25. Diagnosed CKD at age 40.	None.	*IFT140*: p.Y879C	ADPKD
1464	M	37	CGN	Hematuria and proteinuria were noted at age of 15.<br>After that, diagnosed with CKD and hypertension.	None.	*FN1*: p.A1620T	Glomeropathy with fibronectin deposits
1473	M	45	CGN	Proteinuria was noted at age of 35.<br>Diagnosed with hypertension, heart failure	Father has myocardial	*PROK2*: p.R122*	Kallmann syndrome

				at age 41.<br>After that, renal function worsened.	infarction.		
1481	M	34	CGN	Acute nephritis occurred at age 3.<br>Diagnosed with hypertension and AKI at age 29.	Father has AML.	*TSC2*:<br>p.R1122C	TSC
1484	M	43	HNS	Diagnosed with hypertension, chronic heart failure, CKD at age 40.	None.	*SIX5*:<br>p.P316R	BOR syndrome

M, male; F, female, RRT, renal replacement therapy; CGN, chronic glomerulonephritis; PKD, polycystic kidney disease; HNS, hypertensive nephrosclerosis; CKD, chronic kidney disease; NS, nephrotic syndrome; AKI, acute kidney injury; AML, acute myeloid leukemia; ADPKD, autosomal dominant polycystic kidney disease; FSGS, focal segmental glomerular sclerosis; TSC, tuberous sclerosis complex; ADTKD, autosomal dominant tubulointerstitial kidney disease; ADPLD, autosomal dominant polycystic liver disease; BOR, branchio-oto-renal.

Supplementary Table S8. Medical history of patients with truncating variant in genes with an autosomal-recessive inheritance.

Patient N.	Sex	RRT initiation age	Clinical diagnosis	Medical history	Family history	Variants	Genetic diagnosis
1424	M	39	CGN	Diagnosed with CKD at age 35.	None.	*NPHP4*: c.2817+1G>A	NPHP SLSN
1428	F	46	CGN	Diagnosed with CGN at age 13. Diagnosed with CKD when pregnant at age 29.	None.	*IFT172*: p.W205*	NPHP BBS Skeletal ciliopathy
1450	F	44	HNS	Diagnosed with hypertension, proteinuria, CKD at age 23. Diagnosed with preeclampsia at age 32. Suffered from a hypertensive emergency at age 42.	None.	*KIA0586*: p.G15fs	JBTS
1467	M	36	HNS	Hypertension was noted at the age of 34. However, he did not seek medical attention. Diagnosed with heart failure and CKD at age 35.	None.	*WDPCP*: p.Q181fs	BBS

M, male; F, female, RRT, renal replacement therapy; CGN, chronic glomerulonephritis; HNS, hypertensive nephrosclerosis; CKD, chronic kidney disease; NPHP, nephronophthisis; SLSN, Senior Løken syndrome; BBS, Bardet–Biedl syndrome; JBTS, Joubert syndrome.

Supplementary Figure S1. Family trees of patients with pathogenic variants.

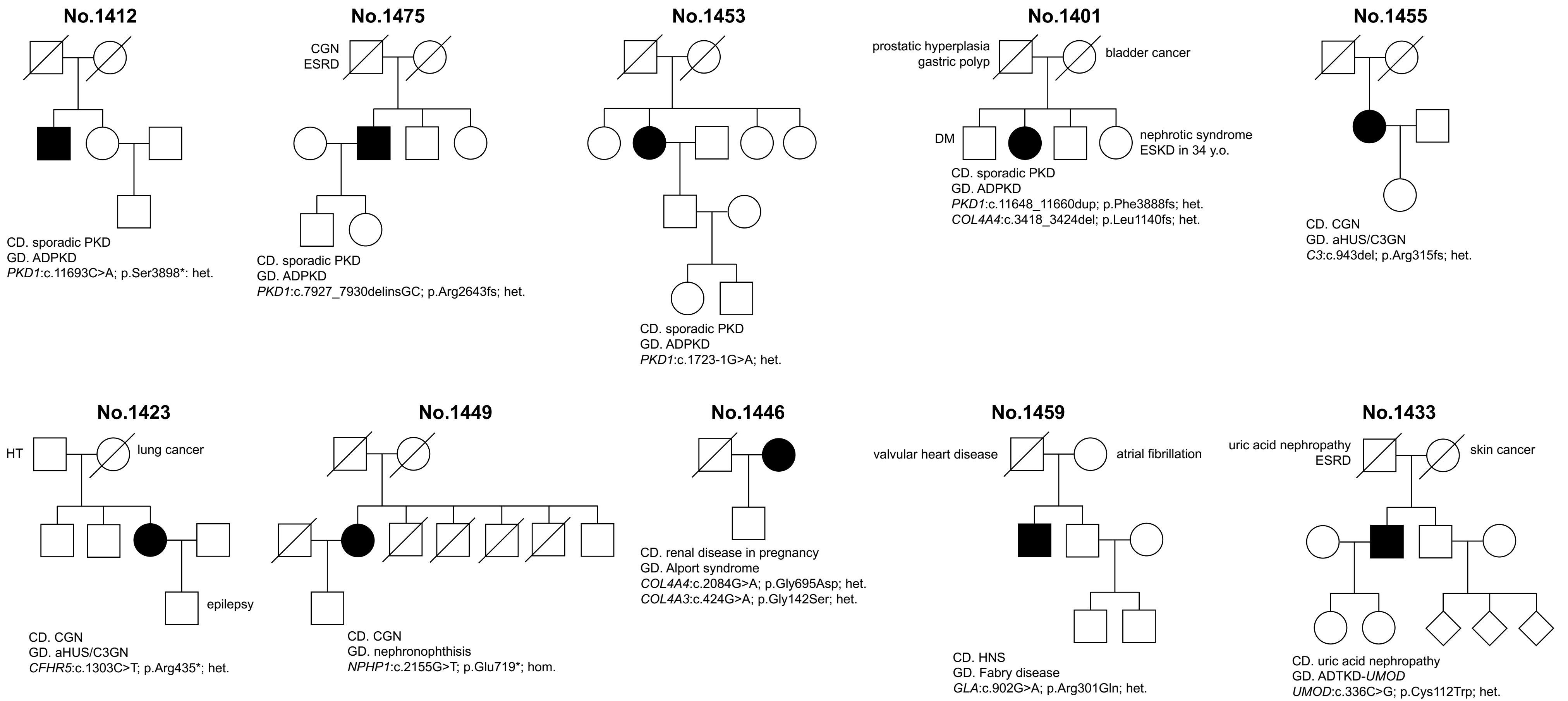

CD, clinical diagnosis; PKD, polycystic kidney disease; GD, genetical diagnosis; ADPKD, autosomal dominant polycystic kidney disease; ESRD, end stage renal disease; DM, diabetic mellitus; CGN, chronic glomerulonephritis; HT, hypertension; aHUS, atypical hemolytic uremic syndrome; C3GN, C3 glomerulopathy; ADTKD, autosomal dominant tubulointerstitial kidney disease.
